# Synergistic effect of sildenafil combined with controlled hypothermia to alleviate microglial activation after neonatal hypoxia–ischemia in rats

**DOI:** 10.1186/s12974-024-03022-w

**Published:** 2024-01-23

**Authors:** Pansiot Julien, Manuela Zinni, Natacha Bonnel, Marina El Kamouh, Felipe Odorcyk, Lea Peters, Emilie-Fleur Gautier, Marjorie Leduc, Cédric Broussard, Olivier Baud

**Affiliations:** 1 Inserm UMR1141 NeuroDiderot, Université Paris Cité, Paris, France; 2 Institut Cochin, Proteom’IC Facility, INSERM, CNRS, Université Paris Cité, Paris, France; 3 Laboratory of Child Growth and Development, University of Geneva, Geneva, Switzerland; 4 Division of Neonatology and Pediatric Intensive Care, Département de Pédiatrie, Hôpitaux Universitaires de Genève, Laboratoire de Développement et Croissance, Children’s University Hospital of Geneva, Geneva, Switzerland

## Abstract

**Background and purpose:**

The only validated treatment to prevent brain damage associated with hypoxia–ischemia (HI) encephalopathy of the newborn is controlled hypothermia with limited benefits. Additional putative neuroprotective drug candidates include sildenafil citrate, a phosphodiesterase-type 5 inhibitor. The main objective of this preclinical study is to assess its ability to reduce HI-induced neuroinflammation, in particular through its potential effect on microglial activation.

**Methods:**

HI was induced in P10 Sprague–Dawley rats by unilateral carotid permanent artery occlusion and hypoxia (HI) and treated by either hypothermia (HT) alone, Sildenafil (Sild) alone or combined treatment (SildHT). Lesion size and glial activation were analyzed by immunohistochemistry, qRT-PCR, and proteomic analyses performed at P13.

**Results:**

None of the treatments was associated with a significant early reduction in lesion size 72h after HI, despite significant changes in tissue loss distribution. Significant reductions in both Iba1 + (within the ipsilateral hemisphere) and GFAP + cells (within the ipsilateral hippocampus) were observed in SildHT group, but not in the other treatment groups. In microglia-sorted cells, pro-inflammatory markers, i.e. Il1b, Il6, Nos2, and CD86 were significantly downregulated in SildHT treatment group only. These changes were restricted to the ipsilateral hemisphere, were not evidenced in sorted astrocytes, and were not sex dependent. Proteomic analyses in sorted microglia refined the pro-inflammatory effect of HI and confirmed a biologically relevant impact of SildHT on specific molecular pathways including genes related to neutrophilic functions.

**Conclusions:**

Our findings suggest that Sildenafil combined with controlled hypothermia produces maximum effect in mitigating microglial activation induced by HI through complex proteomic regulation. The reduction of neuroinflammation induced by Sildenafil may represent an interesting therapeutic strategy for neonatal neuroprotection.

**Supplementary Information:**

111`The online version contains supplementary material available at 10.1186/s12974-024-03022-w.

## Introduction

Neonatal hypoxic-ischemic (HI) encephalopathy following birth asphyxia is a major cause of death or long-term disability in term neonates, affecting about 1–4 per 1000 live births [1]. Incidence and consequences of HI encephalopathy are even more common and severe in less privileged settings, affecting about 1 million infants every year worldwide [2]. In the past 10 years, therapeutic hypothermia (HT) became the standard of care to improve outcomes after perinatal HI insult. Despite HI treatment and state-of-the-art neonatal intensive care, approximately 30% of infants with HI encephalopathy died or developed moderate to severe disability including neurocognitive and epileptic disorders [3–6].

The pathophysiology of perinatal asphyxia in human neonates is mainly related to low cerebral blood flow associated with hypoxemia and early phase reperfusion occurring within the first 6 h after the acute cerebral ischemia. This reperfusion induces systemic and regional hemodynamic disturbances associated with primary energy failure [7]. The second phase of the reperfusion lasting until 48h after injury involves the consequences of excitotoxicity processes and oxidative stress. The later phase beyond 48h includes neuro-inflammatory and brain repair processes extended over several weeks, even under controlled HT [8].

Neuroinflammation likely contributes to the development of long-term neurocognitive impairments [9, 10]. This inflammatory response involves not only neural glial cells, i.e. microglia and astrocytes, but also infiltrating peripheral immune cells such as neutrophils, mast cells and macrophages [11]. While inflammatory response is crucial for promoting repair following an acute brain injury [12–14], some cytokines and other oxidative products can activate deleterious mechanisms that may exacerbate tissue injury [15].

Because controlled HT produces only limited neuroprotective effects [4], additional therapies in combination with HT are currently being investigated. Some recent studies have evaluated the effectiveness of several strategies added to HT, including Erythropoetin, Allopurinol, Xenon, Topiramate, Magnesium Sulphate, and Melatonin [16]. Among these candidates, Sildenafil, a selective inhibitor of phosphodiesterase 5 (PDE5) inducing a potent vasodilation effect by the action on vascular smooth muscle, is commonly used for the treatment of persistent pulmonary hypertension of the newborn [17]. In addition, Sildenafil has been increasingly recognized to confer multiple neuroprotective effects, by acting on the secondary injuries and subsequent repair [18, 19]. In the developing brain, Sildenafil was shown to promote hemodynamic redistribution and vascular density, improving the recruitment of microcirculation [20]. It also reduced brain tissue loss in rodent models of focal cerebral ischemia both early in life [21] and in adulthood [22, 23]. However, neurobiological mechanisms leading to these benefits are still unclear and are likely to involve glial cells.

Here, we asked the question whether Sildenafil may reduce HI-induced neuroinflammation, through its potential regulatory effect on microglial activation, and therefore produce additional benefits to controlled HT in rat pups subjected to HI insult.

## Material and methods

### Ethics statement

All experiments were performed in accordance with the European Community guidelines (Directive 2010/63/EU) and the French National guidelines for the care and use of laboratory animals. All animal procedures were approved by the local Ethics Committee in Animal Experimentation and by the institutional review board (Ministry of Higher Education and Scientific Research, Directorate-General for Research and Innovation, Paris, France (protocol number APAFiS #22114, January 28th 2020).

### Hypoxia–ischemia (HI)

Surgical procedures were performed on P10 Sprague–Dawley rat pups including both sexes (Charles River, Saint Germain Nuelles, France (19.3 ± 1.5 g, n = 239)). Rat pups were randomly divided into 5 groups: Sham controls, untreated HI animals (HI), HI animals treated by hypothermia (HT) alone, Sildenafil (Sild) alone, or by a combination of Sildenafil and Hypothermia (SildHT). On an heating mattress, rat pups underwent a double ligation of the right common carotid artery with 3–0 silk sutures under isoflurane anesthesia (4% for induction and 2% for maintenance). The skin was sutured and the pups were returned to their dams after awakening. After 1h of recovery, the pups were placed in 8% hypoxia during 90 min in a plastic chamber submerged in a water bath at 37 °C, to ensure normothermic environment. Sham controls underwent anesthesia and incision only. All animals were euthanized 72 h after HI (at P13).

### Treatments

**Fig. 1:**
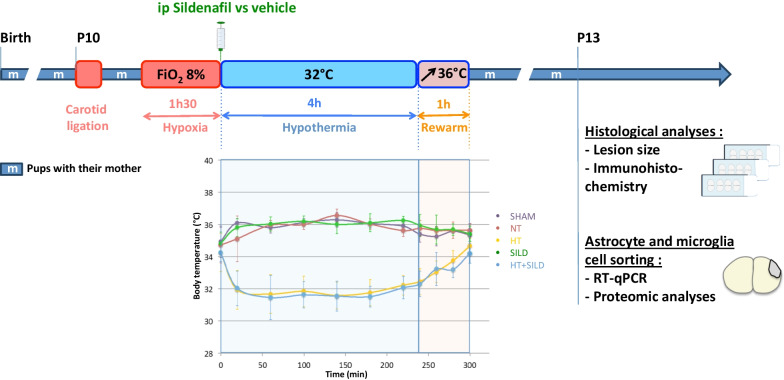
Study design (upper panel) and temperature recordings during controlled hypothermia (n = 45 for the control group, n = 57 for the hypothermia group)

At the end of hypoxia, an intraperitoneal injection of Sildenafil citrate (10 mg/kg, diluted into 5% glucose) was administered on the Sild and SildHT groups pups (injection of the same volume of 5% glucose for the others groups). Afterwards, the HT and SildHT pups were placed into plastic boxes on a hotplate at 25 °C to performed the hypothermia during 4 h (servo-controlled rectal temperature set at 32° ± 1 °C). In the other groups, pups were kept in the plastic chamber submerged in a water bath at 37 °C, to ensure normothermic environment. During the procedure, rectal temperature of all pups was checked every 20–40 min. At the end, a slow rewarming procedure was used with the augmentation of 1 °C of the hotplate every 5 min. Overall study design and body temperature recordings are depicted in the Fig. 1.

### Histological staining and immunohistochemistry

The brains of the rats were collected and immediately placed in 4% formalin solution for 5 days before paraffin embedding. Ten µm coronal paraffin sections were stained with cresyl violet or immunolabeled with primary antibodies: Iba1 (1/500, Wako ref 019-19741, Wako Chemicals, Richmond, VA, USA), GFAP (1/500, Sigma ref G3893, Sigma-Aldrich, St Quentin Fallavier, France) and fluorescent secondary antibodies: Cy3 Donkey Anti-Rabbit IgG (Jackson ImmunoResearch ref 711-165-152, Jackson ImmunoResearch Europe Ldt, Ely, Cambridgeshire, UK) and Alexa Fluor 488 Donkey anti-Mouse IgG (Invitrogen ref A21202, Invitrogen, Carlsbad, CA, USA). For neutrophil infiltration assessment, sections were immunolabeled with a rabbit anti-MPO (1/500, ab208670, Abcam, France) using the NeoStain ABC Kit (Neo-Biotech, NB-23-00005-3). The MPO positive cells were counted in the S1 cortical area.

Total brain tissue loss and hippocampus surface was quantified by calculating the ratio of ipsilateral to contralateral areas measured on cresyl-violet-stained sections. Rostro-caudal measures were performed across all sections of the brain from Bregma + 2.52 mm to − 7.20 mm. For immunohistochemistry, we measured the immunostaining by optical density using the Fiji version of Image [24] in total hemisphere and four delimited brain regions: hippocampus, S1 cortex, perirhinal cortex and thalamus (Additional file 1: Figs. S1, S2). These quantifications were performed on a section nearby the maximal lesion size around Bregman − 3.36 mm. All analyses were performed blinded.

### Magnetic sorting of microglia and astrocyte

Brains were collected from P13 animals and separated in the two hemispheres: ipsilateral (right) and contralateral (left). Primary glial cells were immediately sorted on both using magnetic antibody-based cell sorting (MACS), using CD11b/c antibody (for microglia), as previously reported [25], and antibody ACSA-1 (for astrocytes) (Miltenyi Biotec, Bergisch Gladbach, Germany). Purity of cell sorting was assessed for both microglia and astrocytes as shown in Additional file 1: Fig. S3. Cells were immediately snap frozen after collection for qPCR and proteomic analysis.

### RNA purification, cDNA synthesis, and real-time qPCR

Microglial and astroglial RNA was extracted at P13 using the NucleoSpin RNA Plus XS kit Set (Macherey–Nagel, Hœrdt, France). RNA quantity and quality were determined using the NanodropTM apparatus (Thermofisher Scientific, Waltham, MA, USA). The reverse transcription was performed using the IscriptTM cDNA synthesis kit (Bio-Rad, Marnes-la-Coquette, France), with 500 ng of microglia RNA and 120ng of astrocyte RNA. Primers were designed using Primer3Plus software (primers sequences are described in Additional file 1: Table S1). RT-qPCR was performed in triplicate with the IQ Supermix (Bio-Rad) containing SYBR green, using a 3-step program with 40 cycles. The Ribosomal protein L13 (Rpl13) was used as the reference gene.

### Sample preparation for proteomic analyses

Cell pellets were dissolved in lysis buffer (2% SDS, 100 mM Tris–HCl, pH 8.5) and boiled 5 min at 95 °C. Samples were also reduced and alkylated (10 mM TCEP, 50 mM CAA). Twenty micrograms of each protein extracts were digested using trypsin and an S-Trap Micro Spin Column was used according to the manufacturer’s protocol (Protifi, Farmingdale, NY, USA). Peptides were then speed-vacuum dried.

### Liquid chromatography–coupled mass spectrometry analysis (nLC–MS/MS)

nLC-MS/MS analyses were performed on a Dionex U3000 RSLC nano-LC system coupled to a TIMS-TOF Pro mass spectrometer (Bruker Daltonik GmbH, Bremen, Germany). After drying, peptides were dissolved in 20 μL of 0.1% TFA containing 10% acetonitrile (ACN). Two μL were loaded, concentrated and washed for 3 min on a C18 reverse phase column (5 μm particle size, 100 Å pore size, 300 μm inner diameter, 0.5 cm length, from Thermo-Fisher Scientific). Peptides were separated on an Aurora C18 reverse phase resin (1.6 μm particle size, 100 Å pore size, 75 μm inner diameter, 25 cm length mounted to the Captive nanoSpray Ionisation module, from IonOpticks, Middle Camberwell Australia) with a 4 h run time with a gradient ranging from 98% of solvent A containing 0.1% formic acid in milliQ-grade H_2_O to 35% of solvent B containing 80% acetonitrile, 0.085% formic acid in milliQ-grade H_2_O.

The mass spectrometer acquired data throughout the elution process and operated in DIA PASEF mode with a 1.38 s/cycle, with Timed Ion Mobility Spectrometry (TIMS) enabled and a data-independent scheme with full MS scans in Parallel Accumulation and Serial Fragmentation (PASEF). Ion accumulation and ramp time in the dual TIMS analyzer were set to 100 ms each and the ion mobility range was set from 1/K0 = 0.63 Vs cm^−2^ to 1.43 Vs cm^−2^. Precursor ions for MS/MS analysis were isolated in positive polarity with PASEF in the 400–1.200 m/z range by synchronizing quadrupole switching events with the precursor elution profile from the TIMS device.

### Protein identifications and quantifications

The mass spectrometry data were analyzed using DIA-NN version 1.8.1 [26]. The database used for in silico generation of spectral library was a concatenation of rattus sequences from the Swissprot databases (release 2022-09) and a list of contaminant sequences. Oxydation of methionines was set as variable modification, carbamidomethylation of cysteins was set as permanent modification, and one trypsin misscleavage was allowed. Precursor false discovery rate (FDR) was kept below 1%. The “match between runs” (MBR) option was allowed. The mass spectrometry proteomics data was deposited in the ProteomeXchange Consortium via the PRIDE [27] partner repository with the dataset identifier PXD045168.

### Bioinformatics analyses

For proteomic analyses, sample reproducibility was analyzed by principal component analysis (PCA), performed on Z-score of Log_2_ transformed LFQ values applying a filter of 70% valid values in at least one group (8204/8651 remaining proteins). Quantification analysis was done using home R script. A p-value < 0.01 threshold were used to consider protein differentially significantly expressed. Heat maps were built using R software (version 3.2.3), and clustering was analysed using raw and columns using Euclidean distances on Z-score-transformed values. Volcano plots were generated using R, comparing the differential protein expression between 2 groups. The x-axis of the plots is expression differences (log_2_) and the y-axis is the confidence statistic (− log_10_ p-value).

For between-group comparison (Heat maps and volcano-plots), 2-side unpaired t-test were done on proteins showing at least 2 valid values in each group and 70% of valid values in at least one group using log_2_(LFQ intensity).

An over-representation analysis (ORA) against the Reactome, was performed to identify the enriched Reactome pathways. A sensitivity analysis using ORA geneontology/Biological Process noredundant was also performed. Reactome pathways defined by logF_C_ > 1,3, p-value < 0.05 and FDR < 0.25 were considered significantly up or downregulated.

### Statistical analysis

For RT-qPCR, brain tissue loss and immunofluorescence density, a Mann–Whitney test was performed for the Sham *vs* HI untreated groups comparison and a Kruskal–Wallis test followed by a Dunn’s multiple comparison test to compare HI untreated group and each of the HI treated groups (Sild, HT and SildHT).

Statistical analyses were performed using GraphPad PRISM version 6.0 (GraphPad Software, San Diego, CA). Differences were considered significant at p-value < 0.05. All analyses were performed in a blind setup. Pups from at least 3 litters were used in each experiment.

## Results

**Fig. 2:**
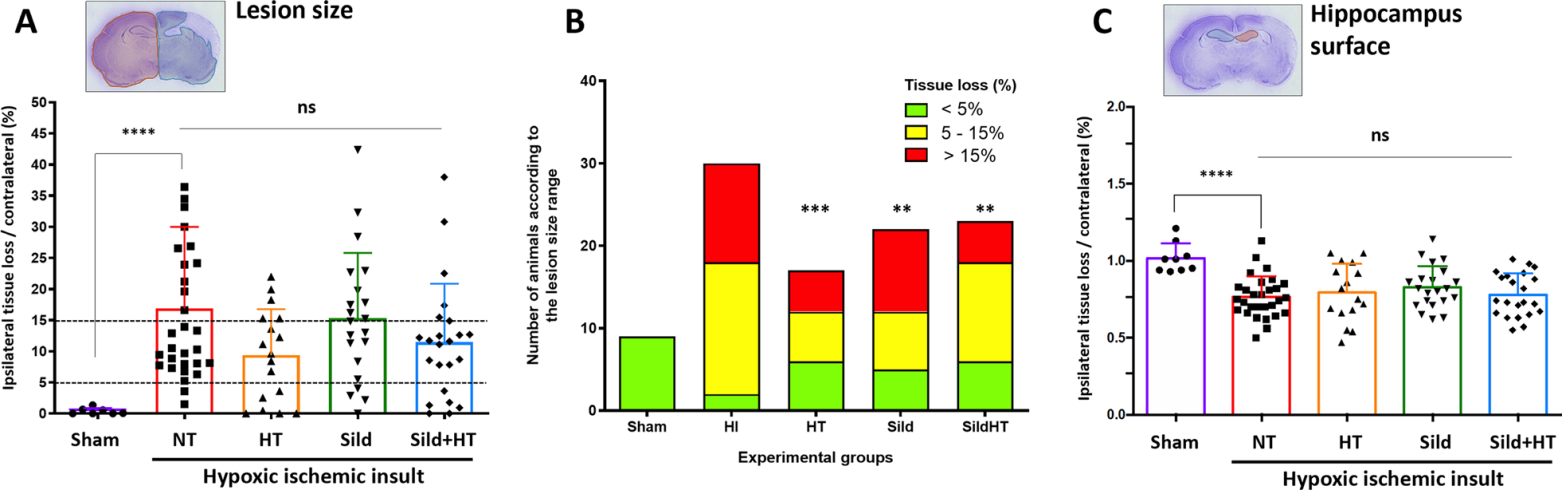
Histological brain lesions in P13 rats subjected to HI at P11 and kept either on normothermia (HI, n = 32) compared to sham (n = 9), or treated by hypothermia alone (HT, n = 17), Sildenafil ip alone (Sild, n = 23), and the combined treatment (Sild + HT, n = 23). Ipsilateral tissue loss (**A**, **B**) and hippocampal surface (C) in right hemisphere were normalized to contralateral hemisphere in Nissl staining. Mean % (SD) of tissue loss (**A**, **C**) or mean (SD) hippocampal surface (**B**) were measured by assessing the entire brain from Bregma + 2.52 mm to − 7.20 mm. In **A** and **C**, sham vs untreated (NT) HI animals were first compared using a non-parametric Mann Whitney t-test (****p < 0.0001). Then, each treatment was compared to untreated animals using a Kruskal–Wallis test (ns: non-significant). In **B**, distribution of lesion size groups was analyzed in each treatment group compared to HI using Chi-square for trend test (***p < 0.001; **p < 0.01)

A highly significant median (IQR) tissue loss of 10.4% (7.8% to 23.3%) in the ipsilateral hemisphere as compared to the contralateral hemisphere was measured 72h after HI (Fig. 2A). None on the treatments tested was associated with significant reduction in lesion size. However, distribution of lesion size among 3 groups (< 5%, 5–15% and > 15% of tissue loss) was found to be significantly different between untreated and treated HI animals (Fig. 2B). In HI animals, ipsilateral hippocampus surface was found to be significantly reduced at P13 by a median (IQR) of 25% (67% to 84%) compared to the contralateral hippocampus, but no treatment was able to prevent this reduction (Fig. 2C).

The reduced weight gain observed in animals subjected to HI, as measured everyday between P10 (baseline) and P13 was found to be similar among treatment groups (Additional file 1: Fig. S4).

**Fig. 3:**
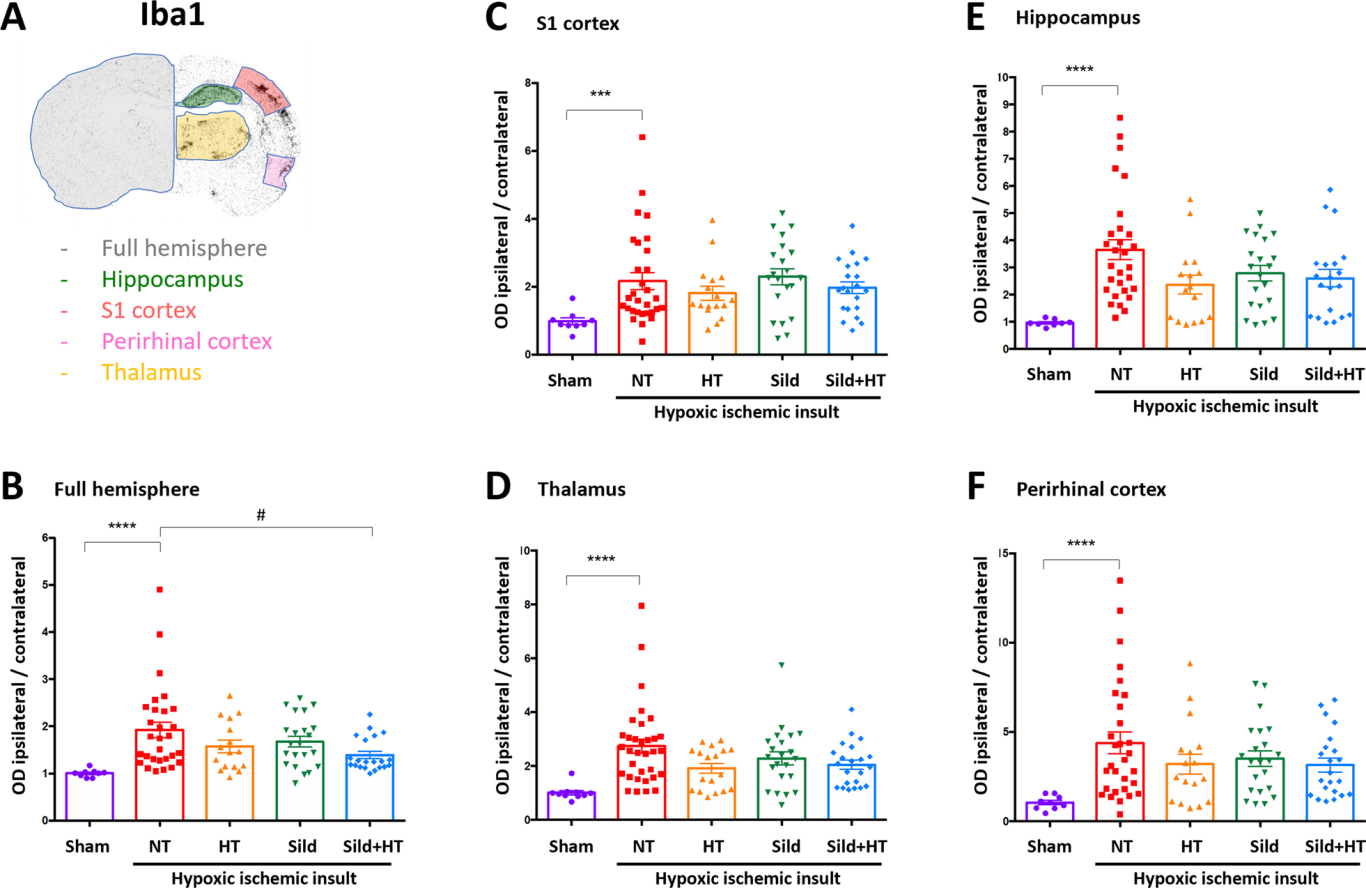
Iba1-positive microglia density in P13 rats subjected to HI at P11 and kept either on normothermia (HI, n = 32) compared to sham (n = 9), or treated by hypothermia alone (HT, n = 17), Sildenafil ip alone (Sild, n = 23), and the combined treatment (Sild + HT, n = 23), in various brain areas. Ipsilateral optical density (OD) of immunoreactive cells was normalized to contralateral hemisphere at Bregma − 3.36 mm. Sham vs untreated (NT) HI animals were first compared using a non-parametric Mann Whitney t-test (***p < 0.001; ****p < 0.0001). Then, each treatment was compared to untreated animals (HI) using a Kruskal–Wallis test followed by a Dunn’s multiple comparison test when appropriate (^#^p < 0.05)

Outside lesion size, we explored the effect of treatments on glial cells density, by using either Iba1 for microglia and GFAP for astrocytes. Both ipsi and contralateral hemispheres were studied as well as several brain areas including those directly damaged by HI (S1 cortex and hippocampus) and adjacent regions of interest (thalamus and perirhinal cortex). HI insult was associated with a highly significant increased density of Iba1-positive (reactive) microglial cells in the ipsilateral hemisphere and specific subregions compared to contralateral areas (Fig. 3). SildHT treatment was found to significantly reduce the microglial density in the overall hemisphere but not in every subregion. Neither HT nor Sild treatment alone was able to significantly change microglial density associated with HI-induced brain damage.

**Fig. 4:**
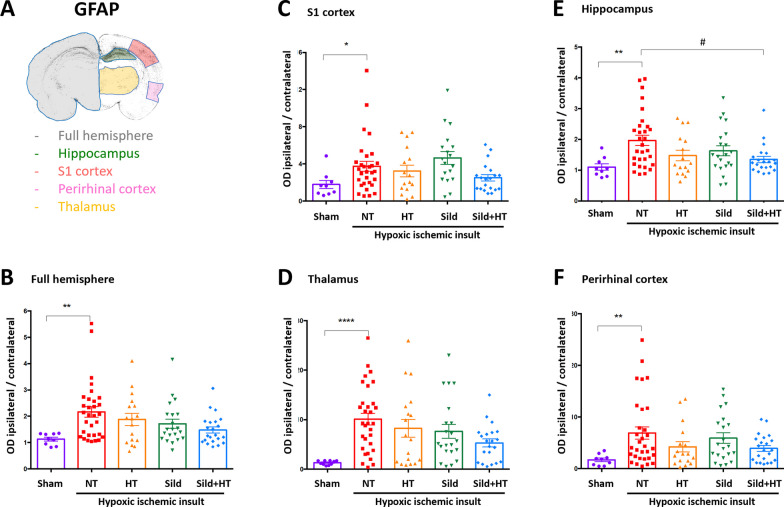
GFAP-positive astrocytes density in P13 rats subjected to HI at P11 and kept either on normothermia (HI, n = 32) compared to sham (n = 9), or treated by hypothermia alone (HT, n = 17), Sildenafil ip alone (Sild, n = 23), and the combined treatment (Sild + HT, n = 23), in various brain areas. Ipsilateral optical density (OD) of immunoreactive cells was normalized to contralateral hemisphere at Bregma − 3.36 mm. Sham vs untreated (NT) HI animals were first compared using a non-parametric Mann Whitney t-test (*p < 0.05; **p < 0.01; ****p < 0.0001). Then, each treatment was compared to untreated animals (HI) using a Kruskal–Wallis test followed by a Dunn’s multiple comparison test when appropriate (^#^p < 0.05)

Similarly, the density of GFAP-positive astrocytes was found to be increased by HI in all conditions (Fig. 4). SildHT treatment significantly reduced astrogliosis only in the hippocampus. All the data were found to be similar between males and females.

**Fig. 5:**
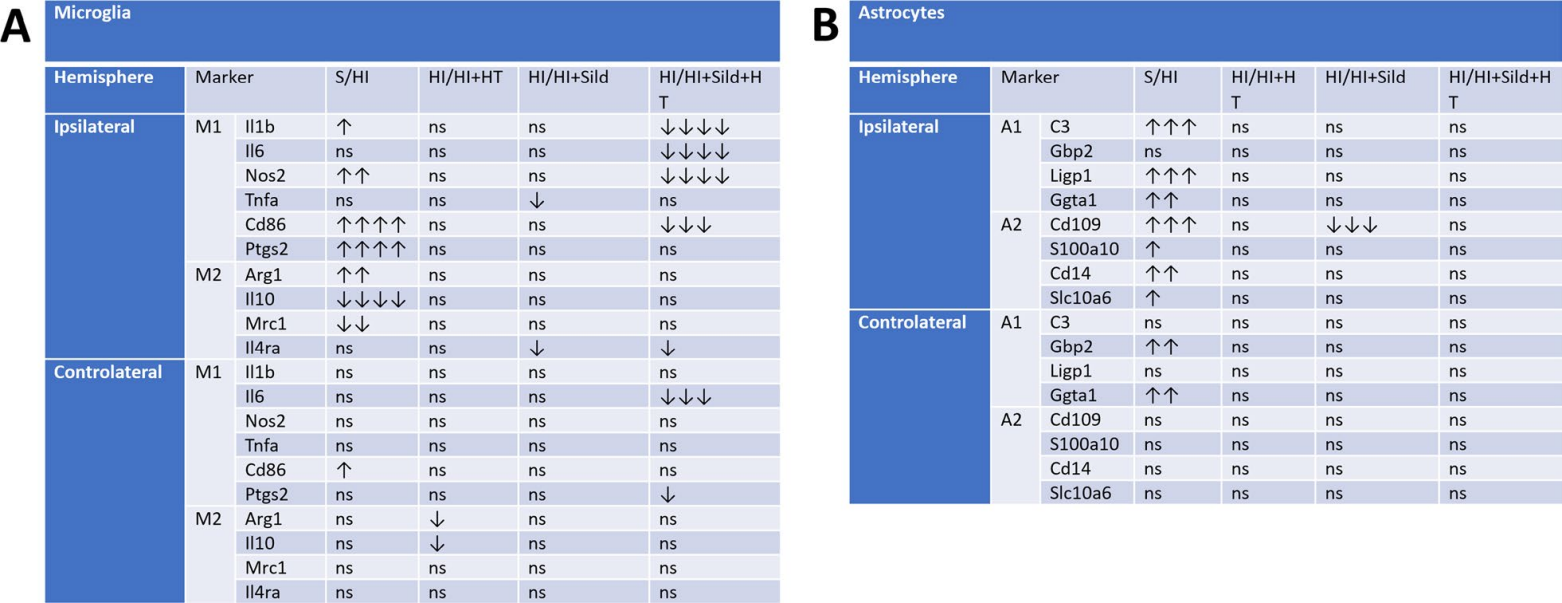
Gene expression polarization in microglial or astroglial cells sorted from P13 rats subjected to HI at P11 and kept either on normothermia (HI, n = 41) compared to sham (n = 12), or treated by hypothermia alone (HT, n = 19), Sildenafil ip alone (Sild, n = 20), and the combined treatment (Sild + HT, n = 18). Data were summarized as changes observed in each category of markers, either in ipsi or contralateral hemisphere. Mann–Whitney test was performed for Sham *vs* HI untreated groups comparison and a Kruskal–Wallis test followed by a Dunn’s multiple comparison test to compare HI untreated group and each of the HI treated groups (Sild, HT and SildHT). Number of arrows corresponds to direction and statistical difference magnitude (one arrow: p < 0.05; 2 arrows: p < 0.01; 3 arrows: p < 0.001; 4 arrows: p < 0.0001; ns: non-significant)

We further studied microglia activation in response to HI with and without treatments by comparing expression of 10 genes previously associated with polarized expression states [28]. This analysis was performed in CD11b-positive cells MACS-sorted from P13 animals of each experimental group and in both hemispheres. In microglia sorted from the ipsilateral hemisphere, HI insult was associated with highly significant increase in most of the pro-inflammatory markers studied including Il1b, Cd86, Nos2, Ptgs2, and Ccl9. Conversely, it induced downregulation of Il10 and Mrc1 genes (Fig. 5A, Additional file 1: Fig. S5). While hypothermia alone and sildenafil alone were associated with no or minimal changes in HI-induced gene expression changes, the combined treatment prevented upregulation of pro-inflammation markers induced by HI, particular Il1b, Nos2, and Cd86, with an additional marginally significant effect on Ptgs2. These findings were not sex-dependent (Additional file 1: Table S2). Very few changes in gene expression were observed in microglia cells sorted from the contralateral hemisphere (Fig. 5A, Additional file 1: Fig. S6).

Similar analyses were performed on ACSA1-positive sorted astrocytes investigating 8 genes involved in astrocytic polarity. Several reactive astrocyte markers of polarization, either A1 (neurotoxic) or A2 (neurotrophic), were found to be increased in response to HI, in particular C3, Ligp1, Ggta1, Cd109 and Cd14, in astrocytes sorted from the ipsilateral hemisphere (Fig. 5B, Additional file 1: Fig. S7). The only treatment effect observed was the downregulation of Cd109 in animals exposed to Sildenafil alone. Only marginal differences in gene expression were detected in the contralateral hemisphere even in response to HI and no treatment effect (Fig. 5B, Additional file 1: Fig. S8).

**Fig. 6:**
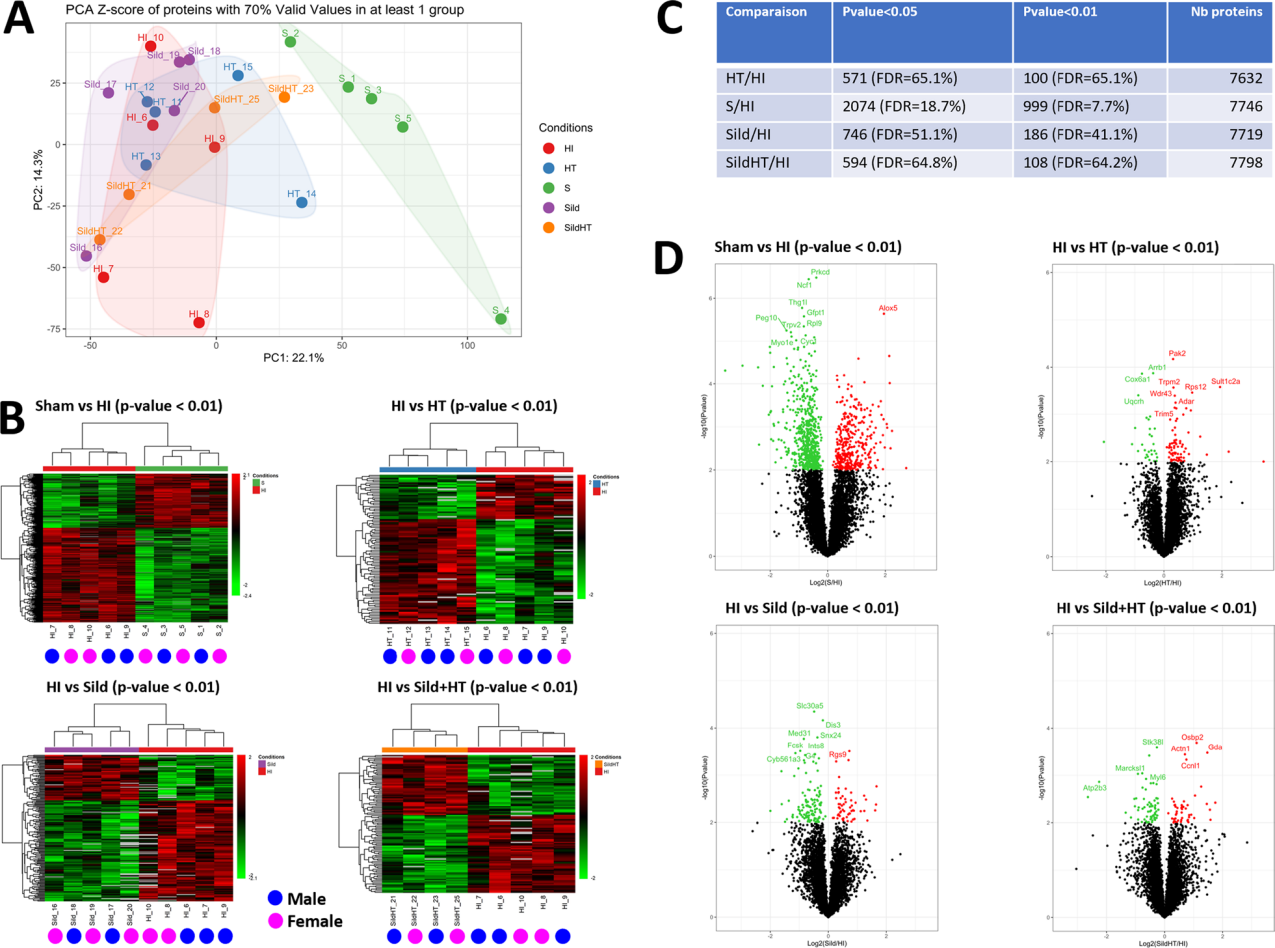
Proteomic analyses of microglial cells sorted from the ipsilateral hemisphere of P13 rats subjected to HI at P11 without and with various neuroprotective treatments (n = 5 in each experimental group). Principal component analysis of the experimental groups (**A**), comparisons between Sham and HI-injured animals and effect of hypothermia alone (HT), Sildenafil ip alone (Sild), and the combined treatment (Sild + HT) (**B**–**D**). For group comparison (Heat maps and volcano-plots), 2-side unpaired t-test were done and a p-value < 0.01 threshold were used to consider protein differentially significantly expressed

Finally, we refined the molecular effects of HI and potential treatments on microglia proteome (for extensive datasets and analyses, see Additional file 1: Tables S3–S10). PCA demonstrated a strong effect of HI compared to Sham animals (Fig. 6A), with limited detectable effect of any treatment. Clustering did not evidence sex-specificity in the up or downregulation of proteins at a p-value < 0.01 in any of the group comparisons (Fig. 6B).

While changes in proteome of sorted microglial from animals subjected to HI had a false discovery rate (FDR) of 8 to 18% when compared to Sham, these FDRs were found to increase from 41 to 65% when HI group was compared to each treatment group (Fig. 6C), findings consistent with the volcano-plots (Fig. 6D). None of these findings was found to be sex-dependent.

**Fig. 7:**
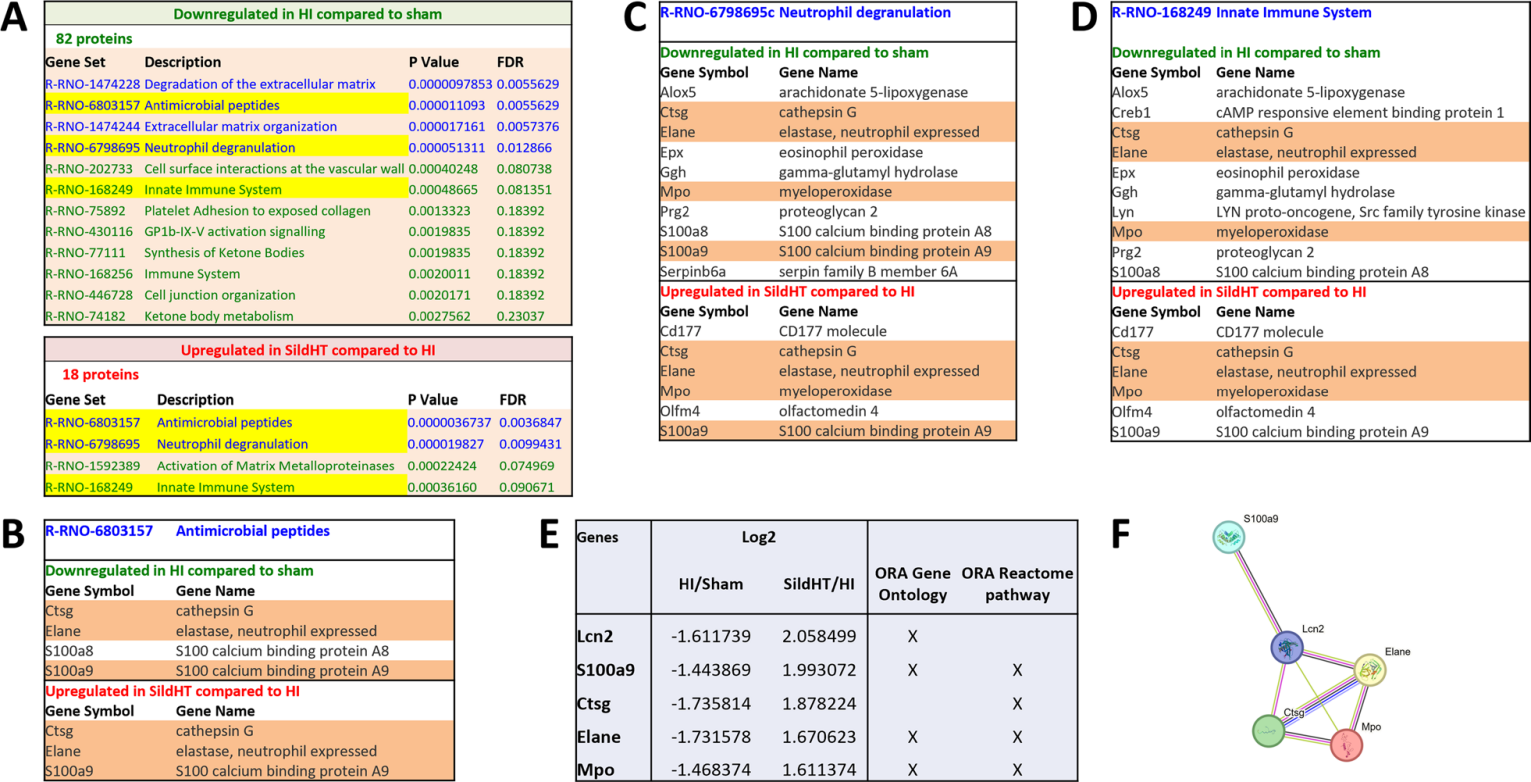
Over-representation analysis (ORA) against the Reactome of the proteome of microglial cells sorted from the ipsilateral hemisphere of P13 rats subjected to HI at P11 without and with neuroprotective treatment Sild + HT. This ORA analysis was performed to identify the enriched Reactome pathways inversely regulated by HI and in HI-injured animals treated by Sild + HT (**A**). Reactome pathways defined by logF_C_ > 1,3, p-value < 0.05 and FDR < 0.25 were considered significantly up or downregulated. Three reactome pathways were identified (**B**–**D**) among them common proteins were also inversely deregulated (highlighted in orange). A sensitivity analysis using ORA geneontology/Biological Process noredundant was also performed. Overall proteins inversely deregulated in each analysis were summarized in **E** and a network of these proteins was obtained using String (http://string-db.org/) with a confidence threshold fixed at 0.4 (medium) (**F**). This network shows significantly more relations than expected by chance (5 nodes, 7 edges while none was expected, p-value = 2.6^–11^)

Both ORA analyses looking for deregulated biological processes or reactome pathways showed important deregulations of many proteins grouped into specific pathways, depending on the statistical threshold used (Additional file 1: Tables S2–S5). No or few changes were detected when HI untreated animals were compared to HT (2 downregulated pathways) or Sild treatment alone (5 up and 1 downregulated). In contrast, up to 13 reactome pathways were found upregulated and 2 downregulated when SildHT animals were compared to HI untreated animals. Similar results were obtained based on GSEA analysis against reactome pathway: as compared to HI untreated animals, no effect of HT alone, limited impact with only 4 reactome inflammation-related pathways downregulated in Sild alone group and up to 30 reactome pathways downregulated by SildHT treatment (Additional file 1: Tables S7–S10). Figure 7 further describes ORA analyses of differentially expressed proteins (p-value < 0.05 at LogFold-change > 1.3) between HI and Sham and then between HI animals treated with HT and Sildenafil and untreated HI animals to identify reactome pathways significantly (FDR < 0.25 and p-value < 0.05) and inversely deregulated in HI and combined treatment groups.

Sixty-two proteins were found to be upregulated in HI group compared to Sham and 82 downregulated. While no upregulated reactome pathways were detected, 12 were found to be significantly downregulated. Among them 3 pathways were inversely deregulated when HI untreated animals were compared to HI animals treated by HT and Sildenafil (Fig. 7A). All proteins included in these 3 pathways (R-RNO-6803157, R-RNO-6798695c, and R-RNO-168249) were further analyzed in Fig. 7B–D. Interestingly, among the proteins significantly downregulated in HI compared to Sham, 4 were found inversely regulated by the combined treatment, both at threshold of logF_C_ > 1,3, p-value < 0.05 and FDR < 0.25, including cathepsin G (Ctsg), Elastase, neutrophil expressed (Elane), Myeloperoxidase (Mpo), and S100 calcium binding protein A9 (S100a9) (Fig. 7E). A sensitivity analysis using GeneOntology/biological process non redundant showed very similar results with 4 genesets inversely regulated by HI and Sild + HT (GO:0009617 response to bacterium; GO:0097237 cellular response to toxic substance; GO:0052547 regulation of peptidase activity; GO:0098542 defense response to other organisms) (Additional file 1: Fig. S9). Four proteins inversely regulated at threshold of logF_C_ > 1,3, p-value < 0.05 and FDR < 0.25 were found in common among these 4 genesets including Elastase, neutrophil expressed (Elane), Lipocalin 2 (Lcn2), Myeloperoxidase (Mpo), and S100 calcium binding protein A9 (S100a9) (Fig. 7E). Network of these proteins obtained using String (http://string-db.org/) with a confidence threshold fixed at 0.4 (medium) is depicted in Fig. 7F. This network shows significantly more relations than expected by chance (5 nodes, 7 edges while none was expected, p-value = 2.6^–11^). Consistently with these proteomic data, immunohistochemistry using anti-MPO antibody revealed a neutrophilic infiltration within the cortical lesion in HI-injured animals highly correlated with the lesion size of corresponding animals (Additional file 1: Fig. S10).

## Discussion

This study is the first describing the molecular changes induced by HI and different treatments in rats in glial cells. We tested controlled HT alone or combined with Sildenafil in a translational approach. While distribution of tissue loss severity was found to be improved by all treatments, improvements in microglial but not astroglial density were evidenced in brain-lesioned animals treated by SildHT only. Both gene expression of specific microglial markers and in-depth proteomic analyses in sorted microglial cells from the injured hemisphere revealed much more significant impact of the combined treatment at a molecular level. In particular, a short list of proteins whose deregulation by HI was found fully prevented by SildHT.

The role of Sildenafil as a promising strategy to prevent or promote repair in adult and neonatal brain was recently reviewed, particularly in cerebral ischemia models [18, 23, 29]. In rodents, Sildenafil acts not only on the immediate deleterious effects induced by the reperfusion, but also on the secondary injuries with reduction of apoptotic cascade, oxidative stress, brain inflammation, and finally on neurorepair by the increase of angiogenesis, neurogenesis and oligodendrogenesis. In the developing brain, Sildenafil was found to reduce infarct size after HI [21, 30], to promote motor function recovery [20, 30, 31], to prevent neuron death or promote neurogenesis [31–34], and enhance oligodendrogenesis [35]. Only a few studies have reported that Sildenafil attenuates reactive gliosis [36] or regulates the activation and polarization of microglia [21]. Here, our findings strongly suggest that, even without a detectable effect on brain lesion size, Sildenafil associated with HT is able to target neuroinflammation by reversing specific pathways deregulated after HI insult in microglial cells.

No significant effect of any treatment tested on infarct size was observed in this study. This is not consistent with previous data showing a significant reduction in lesion size 7 days after HI injury in P7 rats [20] but, here, injury was performed later (P10) and assessment earlier after the insult (72h). In addition, in another mice model focal cerebral ischemia at P9, no significant difference in the brain volume lesions was also observed at 72 h after ischemia in animals exposed to Sildenafil whereas the treatment significantly reduced brain tissue loss 8 days after ischemia [21]. This observation is also relatively in line with a recent multi-drug randomized controlled screening trial in P7 Vannucci model [37] showing either a limited or the lack of effect of controlled hypothermia or Sildenafil respectively, when brain area loss is chosen as the primary outcome. Another difference among studies relates to the acute *vs* chronic exposure to Sildenafil. Here, we have chosen to investigate short-term treatment during early phase of HI insult, with short-term assessment. Yazdani et al. have published complementary studies about chronic exposure to sildenafil given orally to promote repair for 7 days starting 12 h after HI in male pups [30, 32]. Long-term histological assessment at P30 showed significant prevention of HI-induced hippocampal atrophy. Altogether, these data show that neuroprotection based on lesion size may strongly vary according to the animal type, postnatal age at injury and at assessment, and concomitant treatments. Hence, other readouts, i.e. glial activation or its molecular determinants may be much more sensitive and relevant to evidence treatment effect in HI animal models.

This study suggests that molecular changes in microglia appear to be the main effect induced by SildHT treatment in HI animals. Conversely, we found a very limited impact of HI on astrocytes both at immunophenotypic and transcriptomic levels. Immature immune system and biological vulnerability of the developing brain towards oxidative stress lead to an exacerbated neuroinflammatory response to any insult during the neonatal period [38]. Interestingly, the attenuation of microglia reactivity was not necessarily associated with a reduction in the infarct size on a model of focal brain ischemia [36]. We previously reported an effect of Sildenafil alone on both astrogliosis and macrophage/microglial activation 72h and 7 days after HI in P7 Sprague–Dawley rats [20]. Because the present study is the first to investigate the combinatory effect of controlled HT and Sildenafil, we speculate that HT may change glial physiology and may account for cell-specific response to pharmacological treatments. Nevertheless, how this treatment combination may specifically target microglia reactivity and not astrogliosis only needs to be further investigated. Some common pathways whose deregulation by HI were found to be prevented by SildHT in microglia were related to neutrophilic function. Shrivastava et al. have also reported a long-lasting glial response associated with a recruitment of neutrophils from 3h after HI in P7 mice [35]. Among the proteins found in common within these key pathways, neutrophil elastase, myeloperoxidase, cathepsin G, and S100 calcium-binding proteins A9 were found among the most deregulated proteins in human cerebrospinal fluid following severe traumatic brain injury [39].

Translational perspective to test Sildenafil in clinical trials is supported by a recent study showing that a single-dose of Sildenafil improves regional cerebro-vascular deficits in chronic traumatic brain injury [40]. Similarly, Sildenafil increased the event-related sensory and visual BOLD response compared with placebo in adult patients with Becker muscular dystrophy [41]. These findings also demonstrate that Sildenafil is able to efficiently cross the blood brain barrier (BBB). A recent clinical trial (SANE study, NCT02812433) has investigated (i) whether oral Sildenafil given from day 2 to day 9, can be safely used in asphyxiated newborns treated with hypothermia, (ii) pharmacokinetics and pharmacodynamics profiles in newborn treated by controlled HT, and (iii) its effect on brain injury on day 30 of life. A large multicenter randomized clinical trial (SHINE trial) is planned to recruit neonates with HIE with several foreseeable benefits including increased survival rate, lower incidence of brain damage, lower risk of neurodevelopmental impairments in surviving patients, and reduction in the need for post-neonatal intervention to prevent and/or treat neurocognitive disorders.

The main strengths of this preclinical study include the random allocation of animals to treatment groups, blinded assessments, in depth analyses of glial sorted cells, and large sample size in each treatment group. This study also has some limitations related to the its pre-determined short-term outcomes. Indeed, we decided to investigate the effect of each treatment on lesion size and in glial cells only. Therefore, functional consequences of molecular changes observed in HI-induced microglial activation remain speculative. Second, while infiltration of peripheral immune cells into the brain parenchyma is tightly regulated by the BBB in basal conditions [42], they may colonize the injured brain after HI-induced BBB disruption [43]. CD11b/c cell sorting is not able to discard peripheral immune cells but microglia were found to be the most prominent cell type in rat pups, as previously reported [44]. Third, analyses were performed at a single time point, precluding any assessment of the time-depended changes in inflammation induced by the models and possible delayed treatment effects. Indeed, in mice, HT was recently found to significantly reduce HI-induced brain injury after 7 days post insult, whereas acute suppression of HI-induced upregulation of inflammatory genes was observed earlier in myeloid cells and decreased infiltration of peripheral macrophages [45]. Hence, we cannot rule out our data may underestimate the effect size of the treatments at later stages of the inflammation and repair processes.

In conclusion, this study provides novel insights supporting the benefit of Sildenafil citrate associated with HT to mitigate microglial activation induced by HI. This regulation was consistently observed at both immunohistochemical, transcriptomic and proteomic levels. Sildenafil may represent a promising therapeutic strategy for neonatal neuroprotection in combination with HT.

### Supplementary Information

**Additional file 1: Figure S1.** Segmentation of the brain slices according to Paxinos and Watson Atlas (1998)* used for immunohistochemistry. Quantifications were performed on a section nearby the maximal lesion size around Bregman -3.36mm. * Paxinos G and Watson C. 1998. The rat brain in stereotaxic coordinates, 4th ed. San Diego, CA, USA; Academic press. **Figure S2**. Quantification of cell-specific staining within segmented brain area, from a standardized threshold. Imaging processing for assessing immunostaining using the Fiji software in total hemisphere and four delimited brain regions: hippocampus, S1 cortex, perirhinal cortex and thalamus. Quantifications were performed after images were converted into black and white 8-bit signal and according to a predefined standardized threshold. **Figure S3. **Assessment of cell purity after magnetic antibody-based cell sorting. A: CD11b/c positive cells showed a large majority of Cx3cr1positive cells and less than 1% astrocytic contamination. B: ACSA-1 positive cells showed a large proportion of GFAP positive astrocytes and less than 5% contamination from oligodendroglial lineage. RDC means recurrent DNA double-strand break clusters observed in neural stem/progenitor cells. **Figure S4.** Body weight gain between P10 and P13 rats subjected to HI at P11 and kept either on normothermia (HI, n = 78) compared to sham (n = 26), or treated by hypothermia alone (HT, n = 41), Sildenafil ip alone (Sild, n = 48), and the combined treatment (Sild + HT, n = 46). Body weight changes for each day compared to P10 are expressed mean ± SD. Weight gain in Sham group (controls) was compared in each experimental groups using one-way ANOVA with Dunnet’s multiple comparisons test (***: p < 0.001; ****: p < 0.0001). **Figure S5.** Gene expression polarization in Iba1-positive MG/Mf cells sorted from the ipsilateral hemisphere of P13 rats subjected to HI at P11 and kept either on normothermia (HI, n = 41) compared to sham (n = 12), or treated by hypothermia alone (HT, n = 19), Sildenafil ip alone (Sild, n = 20), and the combined treatment (Sild + HT, n = 18). Detailed gene expression of several pro-inflammatory (A) and immunoregulatory/anti-inflammatory markers (B). Quantified results are mean ± SD. Sham vs untreated (NT) HI animals were first compared using a non-parametric Mann Whitney t-test (*: p < 0.05; **: p < 0.01; ****: p < 0.0001). Then, each treatment was compared to untreated animals (HI) using a Kruskal–Wallis test followed by a Dunn’s multiple comparison test when appropriate (#: p < 0.05; ###: p < 0.001; ####: p < 0.0001). **Figure S6.** Gene expression polarization in Iba1-positive MG/Mf cells sorted from the contralateral hemisphere of P13 rats subjected to HI at P11 and kept either on normothermia (HI, n = 41) compared to sham (n = 12), or treated by hypothermia alone (HT, n = 19), Sildenafil ip alone (Sild, n = 20), and the combined treatment (Sild + HT, n = 18). Detailed gene expression of several pro-inflammatory (A) and immunoregulatory/anti-inflammatory markers (B). Quantified results are mean ± SD. Sham vs untreated (NT) HI animals were first compared using a non-parametric Mann Whitney t-test (*: p < 0.05). Then, each treatment was compared to untreated animals (HI) using a Kruskal–Wallis test followed by a Dunn’s multiple comparison test when appropriate (#: p < 0.05; ###: p < 0.001; ####: p < 0.0001). **Figure S7.** Gene expression polarization in GFAP-positive astrocytes sorted from the ipsilateral hemisphere of P13 rats subjected to HI at P11 and kept either on normothermia (HI, n = 41) compared to sham (n = 12), or treated by hypothermia alone (HT, n = 19), Sildenafil ip alone (Sild, n = 20), and the combined treatment (Sild + HT, n = 18). Detailed gene expression of A1 (A) and A2 astrocytic markers (B). Quantified results are mean ± SD. Sham vs untreated (NT) HI animals were first compared using a non-parametric Mann Whitney t-test (*: p < 0.05; **: p < 0.01; ***: p < 0.001). Then, each treatment was compared to untreated animals (HI) using a Kruskal–Wallis test followed by a Dunn’s multiple comparison test when appropriate (###: p < 0.001). **Figure S8.** Gene expression polarization in GFAP-positive astrocytes sorted from the contralateral hemisphere of P13 rats subjected to HI at P11 and kept either on normothermia (HI, n = 41) compared to sham (n = 12), or treated by hypothermia alone (HT, n = 19), Sildenafil ip alone (Sild, n = 20), and the combined treatment (Sild + HT, n = 18). Detailed gene expression of A1 (A) and A2 astrocytic markers (B). Quantified results are mean ± SD. Sham vs untreated (NT) HI animals were first compared using a non-parametric Mann Whitney t-test (**: p < 0.01). Then, each treatment was compared to untreated animals (HI) using a Kruskal–Wallis test followed by a Dunn’s multiple comparison test (all comparisons are not significant). **Figure S9.** Over-representation analysis (ORA) using GeneOntology/biological process non redundant of the proteome of MG/Mf cells sorted from the ipsilateral hemisphere of P13 rats subjected to HI at P11 without and with neuroprotective treatment Sild + HT. This ORA analysis was performed to identify the enriched genesets inversely regulated by HI and in HI-injured animals treated by Sild + HT. GO pathways defined by logFC > 1,3, p-value < 0.05 and FDR < 0.25 were considered significantly up or downregulated. Four genesets were identified as inversely deregulated (highlighted in yellow). **Figure S10.** Neutrophilic infiltration in the lesion site. Quantification of MPO + cells within S1 cortical brain area, and typical pictures of labelled cells in Sham and HI animals (A). Data are expressed as mean ± SD. Sham vs untreated (NT) HI animals were compared using a non-parametric Mann Whitney t-test (*: p < 0.05). Linear regression between lesion size and MPO + cells density in each animals (B). Table S1: Primers sequences used for RT-qPCR. **Table S2.** Analyses of main endpoints according to sex and interaction between sex and each variable analyzed. Quantified results are mean ± SD or SEM. Sham vs untreated (NT) HI animals were first compared using a non-parametric Mann Whitney t-test (*: p < 0.05; **: p < 0.01; ***: p < 0.001; ****: p < 0.0001). Then, each treatment was compared to untreated animals (HI) using a Kruskal–Wallis test followed by a Dunn’s multiple comparison test when appropriate (#: p < 0.05; ##:p < 0.01; ###: p < 0.001). Two-way ANOVA was used to assess interaction between sex and treatment. **Table S3.** ORA analyses looking for deregulated biological processes in the HI group compared to the Sham group, depending on various the statistical thresholds. **Table S4.** ORA analyses looking for deregulated biological processes in the HI + hypothermia group (HT) compared to the HI untreated group, depending on various the statistical thresholds. **Table S5.** ORA analyses looking for deregulated biological processes in the HI + Sildenafil group (Sild) compared to the HI untreated group, depending on various the statistical thresholds. **Table S6.** ORA analyses looking for deregulated biological processes in the HI + Hypothermia + Sildenafil group (SildHT) compared to the HI untreated group, depending on various the statistical thresholds. **Table S7.** GSEA analysis against reactome pathways looking for deregulated biological processes in the HI untreated group compared to the Sham group. **Table S8.** GSEA analysis against reactome pathways looking for deregulated biological processes in the HI + Hypothermia group compared to the HI untreated group. **Table S9.** GSEA analysis against reactome pathways looking for deregulated biological processes in the HI + Sildenafil group compared to the HI untreated group. **Table S10.** GSEA analysis against reactome pathways looking for deregulated biological processes in the HI + Hypothermia + Sildenafil group compared to the HI untreated group.

## Data Availability

Proteomics data are available in the ProteomeXchange Consortium with the dataset identifier PXD045168. All other data are fully available upon request.
