# Supplementary material for: Synergistic effect of sildenafil combined with controlled hypothermia to alleviate microglial activation after neonatal hypoxia–ischemia in rats
Source: J Neuroinflammation. 2024 Jan 23;21:31. doi: 10.1186/s12974-024-03022-w (PMC10804557; doi:10.1186/s12974-024-03022-w)
Supplement: Supplementary file 1 — Additional file 1: Figure S1. Segmentation of the brain slices according to Paxinos and Watson Atlas (1998)* used for immunohistochemistry. Quantifications were performed on a section nearby the maximal lesion size around Bregman -3.36mm. * Paxinos G and Watson C. 1998. The rat brain in stereotaxic coordinates, 4th ed. San Diego, CA, USA; Academic press. Figure S2. Quantification of cell-specific staining within segmented brain area, from a standardized threshold. Imaging processing for assessing immunostaining using the Fiji software in total hemisphere and four delimited brain regions: hippocampus, S1 cortex, perirhinal cortex and thalamus. Quantifications were performed after images were converted into black and white 8-bit signal and according to a predefined standardized threshold. Figure S3. Assessment of cell purity after magnetic antibody-based cell sorting. A: CD11b/c positive cells showed a large majority of Cx3cr1positive cells and less than 1% astrocytic contamination. B: ACSA-1 positive cells showed a large proportion of GFAP positive astrocytes and less than 5% contamination from oligodendroglial lineage. RDC means recurrent DNA double-strand break clusters observed in neural stem/progenitor cells. Figure S4. Body weight gain between P10 and P13 rats subjected to HI at P11 and kept either on normothermia (HI, n = 78) compared to sham (n = 26), or treated by hypothermia alone (HT, n = 41), Sildenafil ip alone (Sild, n = 48), and the combined treatment (Sild + HT, n = 46). Body weight changes for each day compared to P10 are expressed mean ± SD. Weight gain in Sham group (controls) was compared in each experimental groups using one-way ANOVA with Dunnet’s multiple comparisons test (***: p < 0.001; ****: p < 0.0001). Figure S5. Gene expression polarization in Iba1-positive MG/Mf cells sorted from the ipsilateral hemisphere of P13 rats subjected to HI at P11 and kept either on normothermia (HI, n = 41) compared to sham (n = 12), or treated by hypothermia a [file 12974_2024_3022_MOESM1_ESM.pdf]

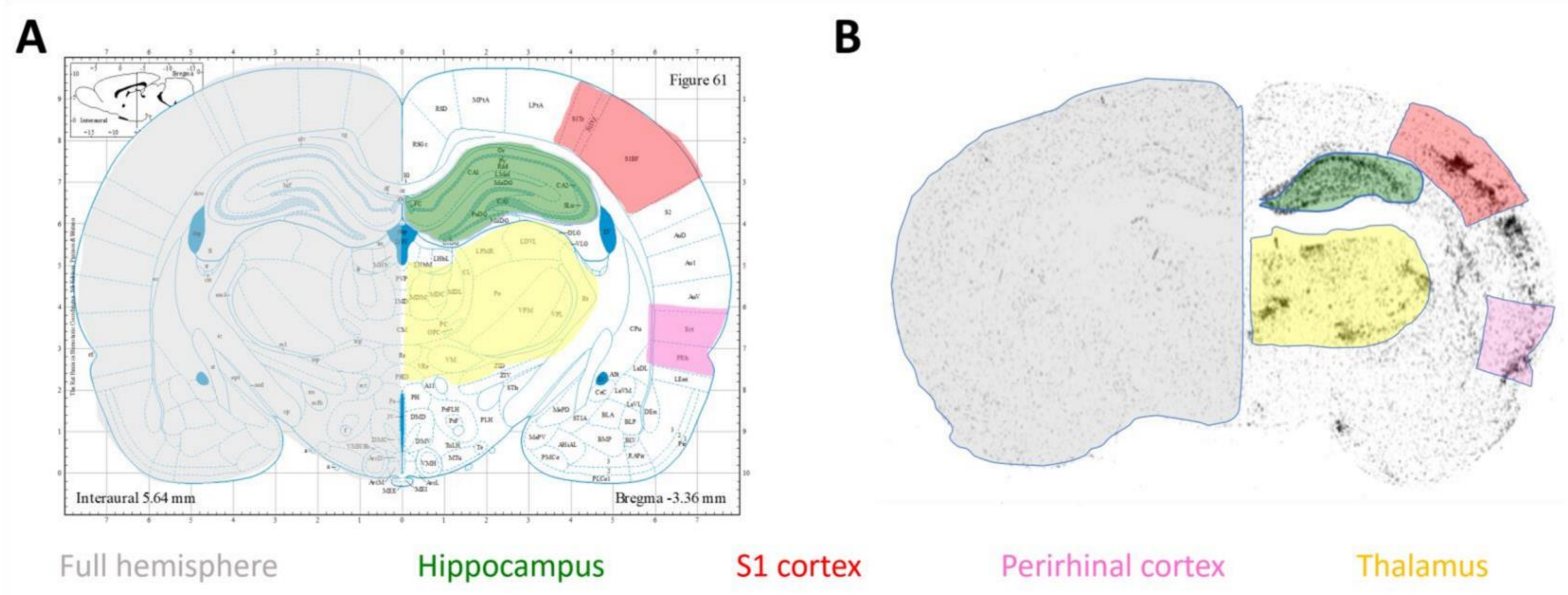

Figure S1: Segmentation of the brain slices according to Paxinos and Watson Atlas (1998)* used for immunohistochemistry. Quantifications were performed on a section nearby the maximal lesion size around Bregman -3.36mm.

* Paxinos G and Watson C. 1998. The rat brain in stereotaxic coordinates, 4th ed. San Diego, CA, USA; Academic press.

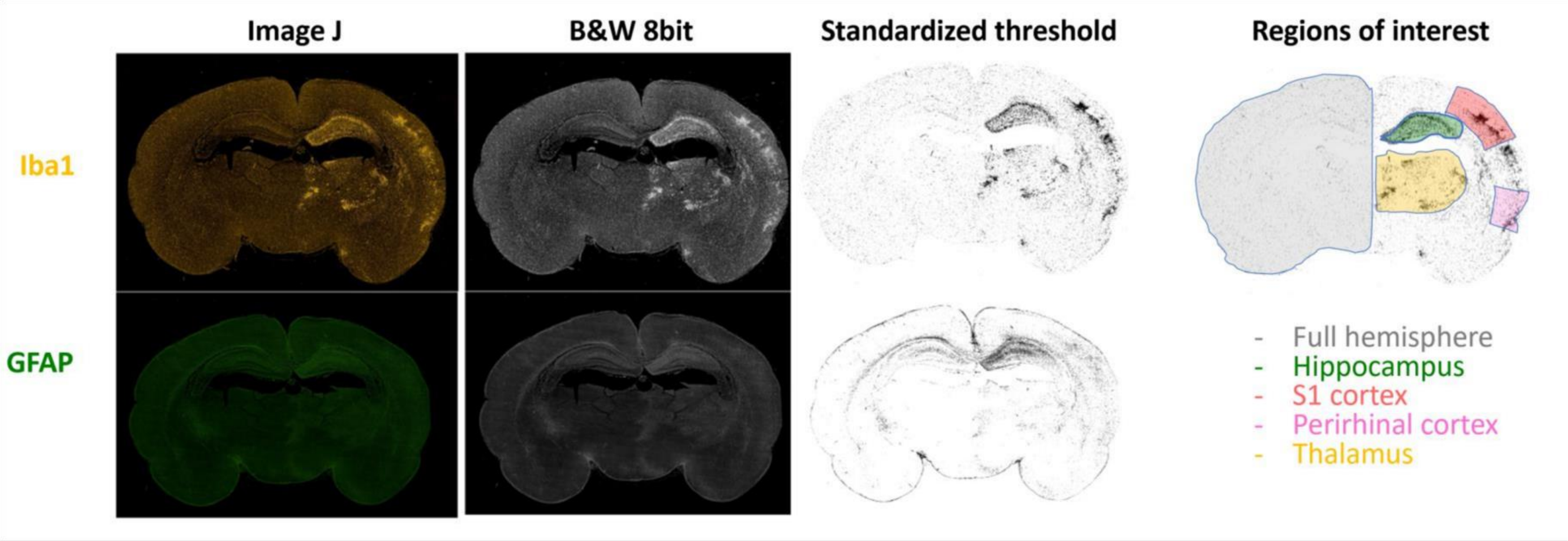

Figure S2: Quantification of cell-specific staining within segmented brain area, from a standardized threshold. Imaging processing for assessing immunostaining using the Fiji software in total hemisphere and four delimited brain regions: hippocampus, S1 cortex, perirhinal cortex and thalamus.

Quantifications were performed after images were converted into black and white 8-bit signal and according to a predefined standardized threshold.

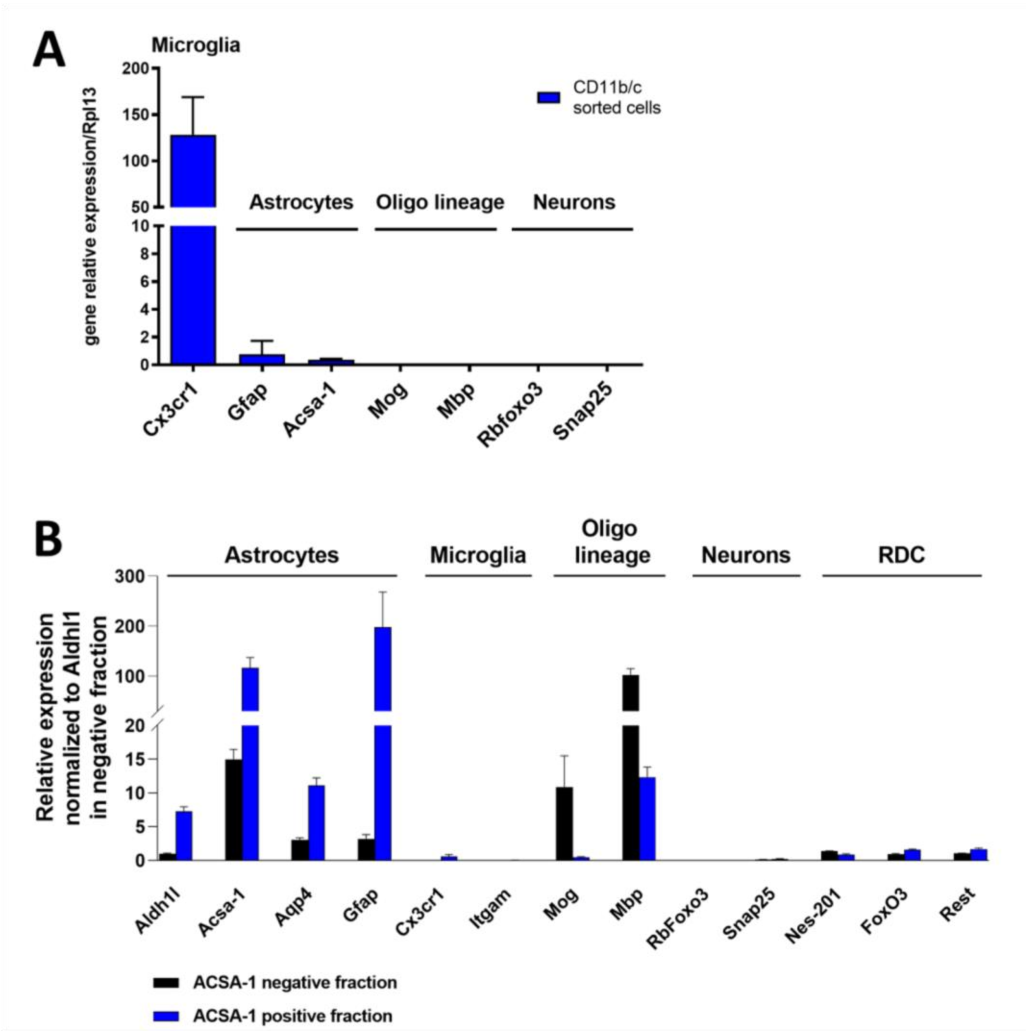

Figure S3: Assessment of cell purity after magnetic antibody-based cell sorting. A: CD11b/c positive cells showed a large majority of Cx3CR1-positive cells and less than 1% astrocytic contamination. **B:** ACSA-1 positive cells showed a large proportion of GFAP positive astrocytes and less than 5% contamination from oligodendroglial lineage. RDC means recurrent DNA double-strand break clusters observed in neural stem/progenitor cells.

	SHAM	HI	HI+HT	HI+SILD	HI+SILD+HT
P11	2.66±0.17	0.68±0.17****	0.73±0.31***	0.67±0.21***	0.19±0.29****
P12	4.56±0.24	2.45±0.20****	2.49±0.37***	2.55±0.24***	1.53±0.34****
P13	5.67±0.36	3.63±0.21****	3.76±0.40***	4.04±0.26***	3.00±0.33****

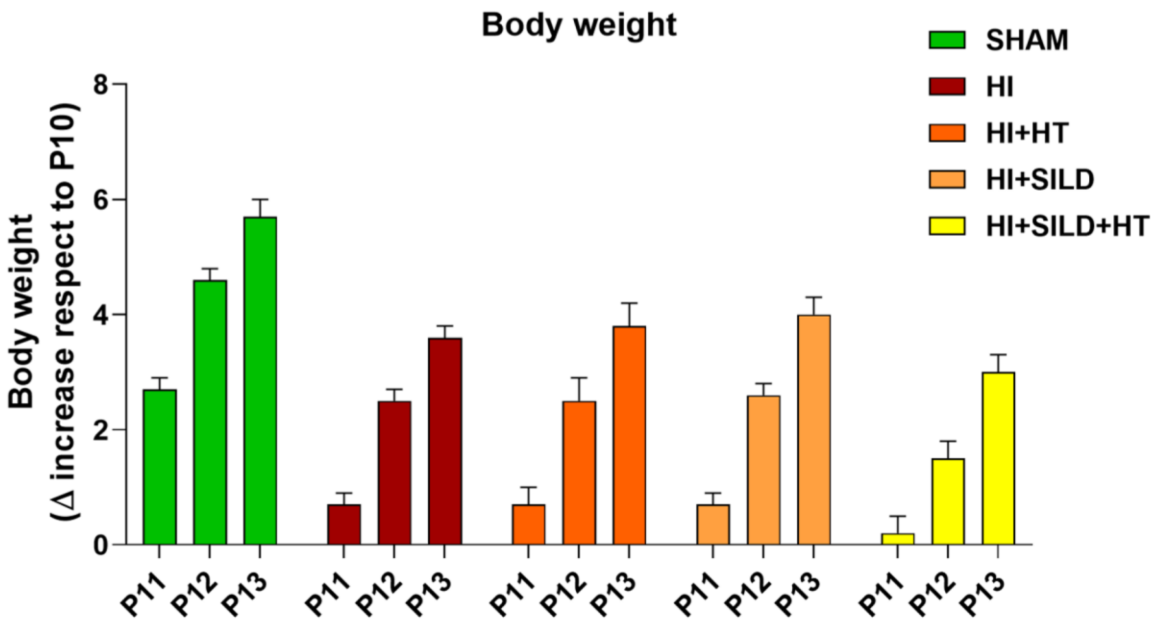

Figure S4: Body weight gain between P10 and P13 rats subjected to HI at P11 and kept either on normothermia (HI, n=78) compared to sham (n=26), or treated by hypothermia alone (HT, n=41), Sildenafil ip alone (Sild, n=48), and the combined treatment (Sild+HT, n=46). Body weight changes for each day compared to P10 are expressed in mean ± SEM. Weight gain in Sham group (controls) was compared in each experimental groups using one-way ANOVA with Dunnet's multiple comparisons test (***: p<0.001; ****: p<0.0001).

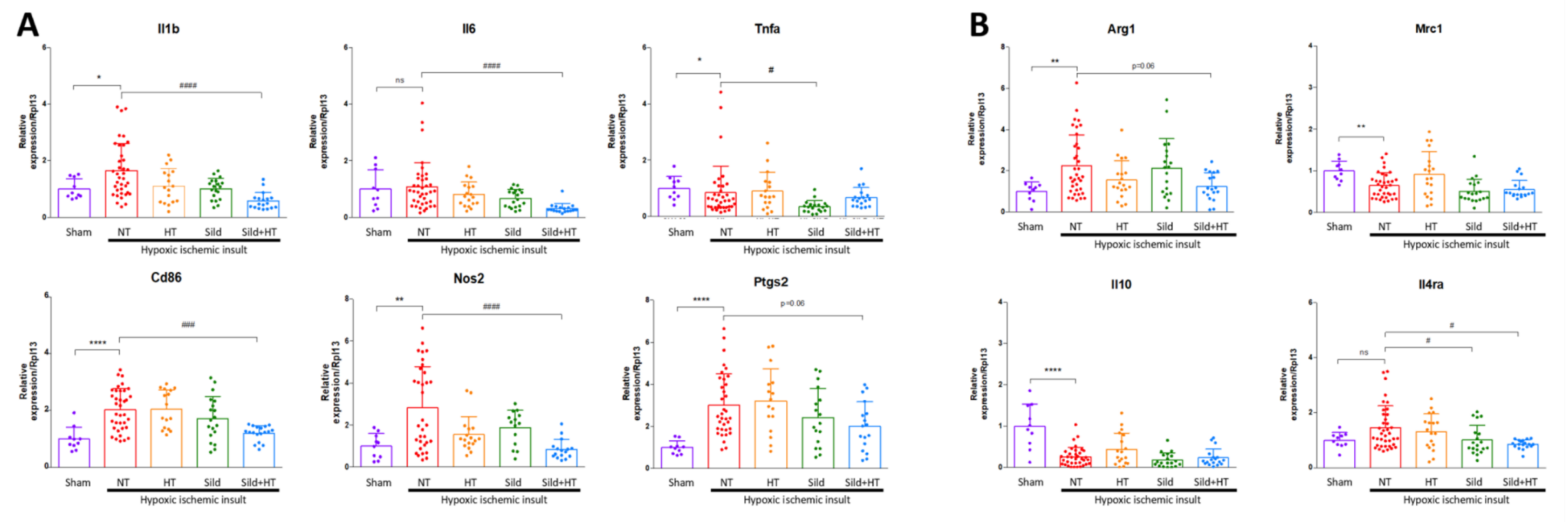

Figure S5: **Gene expression polarization in Iba1-positive MG/M□ cells sorted from the ipsilateral hemisphere of P13 rats subjected to HI at P11 and kept either on normothermia (HI, n=41) compared to sham (n=12), or treated by hypothermia alone (HT, n=19), Sildenafil ip alone (Sild, n=20), and the combined treatment (Sild+HT, n=18).** Detailed gene expression of several pro-inflammatory (A) and immunoregulatory/anti-inflammatory markers (B). Quantified results are mean ± SD. Sham vs untreated (NT) HI animals were first compared using a non-parametric Mann Whitney t-test (*: p<0.05; **: p<0.01; ****: p<0.0001). Then, each treatment was compared to untreated animals (HI) using a Kruskal-Wallis test followed by a Dunn's multiple comparison test when appropriate (#: p<0.05; ###: p<0.001; ####: p<0.0001).

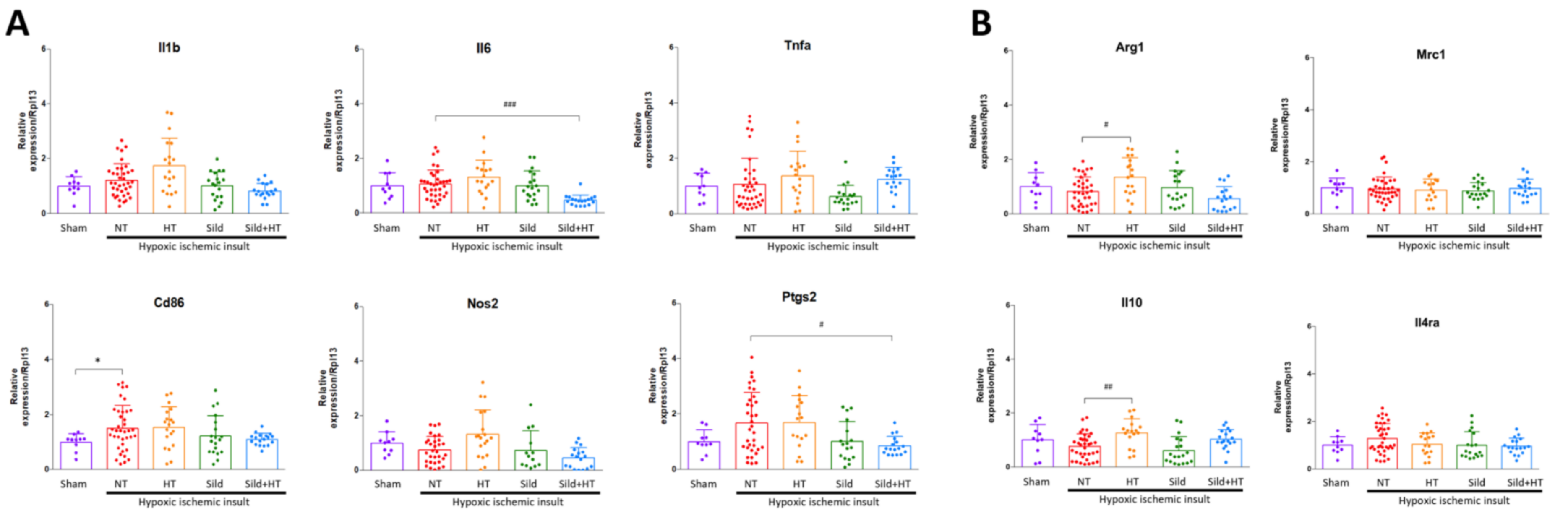

Figure S6: Gene expression polarization in Iba1-positive MG/M□ cells sorted from the contralateral hemisphere of P13 rats subjected to HI at P11 and kept either on normothermia (HI, n=41) compared to sham (n=12), or treated by hypothermia alone (HT, n=19), Sildenafil ip alone (Sild, n=20), and the combined treatment (Sild+HT, n=18). Detailed gene expression of several pro-inflammatory (A) and immunoregulatory/anti-inflammatory markers (B). Quantified results are mean ± SD. Sham vs untreated (NT) HI animals were first compared using a non-parametric Mann Whitney t-test (*: $p<0.05$). Then, each treatment was compared to untreated animals (HI) using a Kruskal-Wallis test followed by a Dunn's multiple comparison test when appropriate (#: $p<0.05$; ###: $p<0.001$; ####: $p<0.0001$).

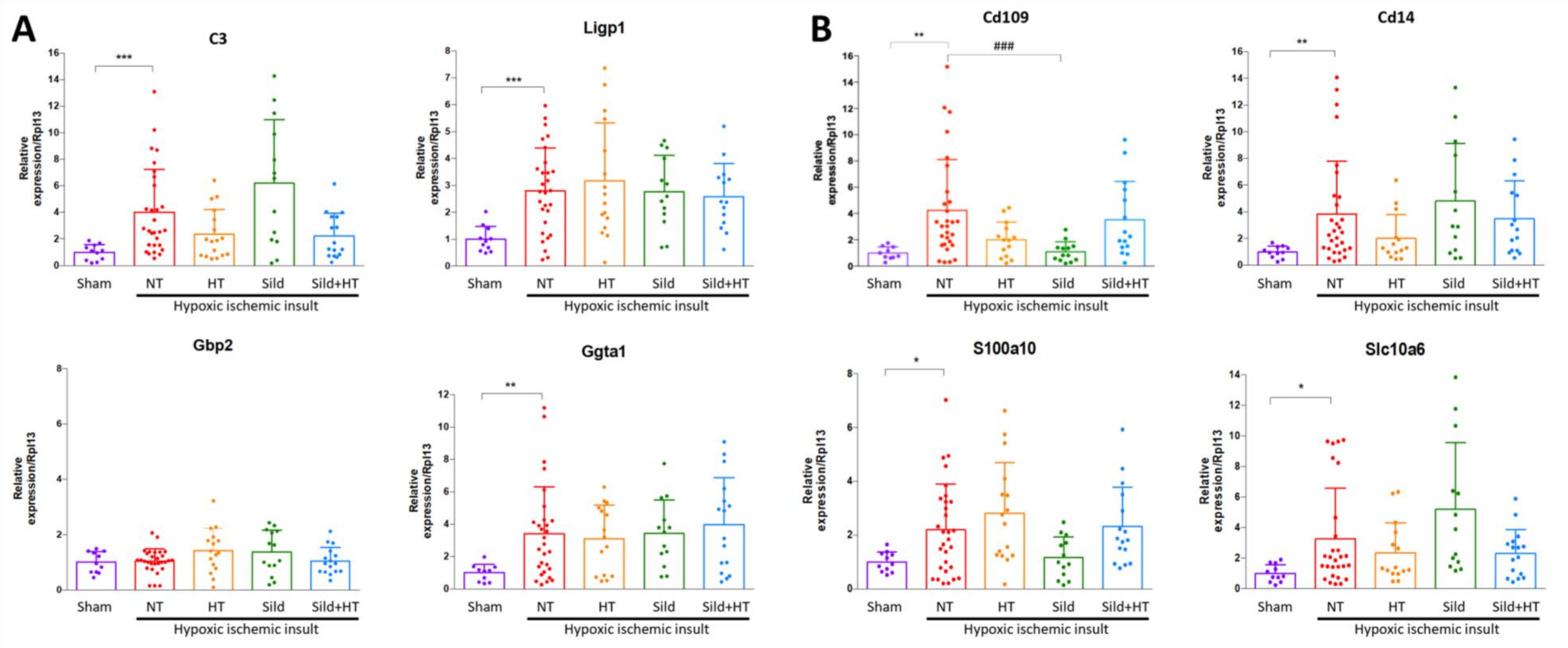

Figure S7: Gene expression polarization in GFAP-positive astrocytes sorted from the ipsilateral hemisphere of P13 rats subjected to HI at P11 and kept either on normothermia (HI, n=41) compared to sham (n=12), or treated by hypothermia alone (HT, n=19), Sildenafil ip alone (Sild, n=20), and the combined treatment (Sild+HT, n=18). Detailed gene expression of A1 (A) and A2 astrocytic markers (B). Quantified results are mean ± SD. Sham vs untreated (NT) HI animals were first compared using a non-parametric Mann Whitney t-test (*: $p<0.05$; **: $p<0.01$; ***: $p<0.001$). Then, each treatment was compared to untreated animals (HI) using a Kruskal-Wallis test followed by a Dunn's multiple comparison test when appropriate (###: $p<0.001$).

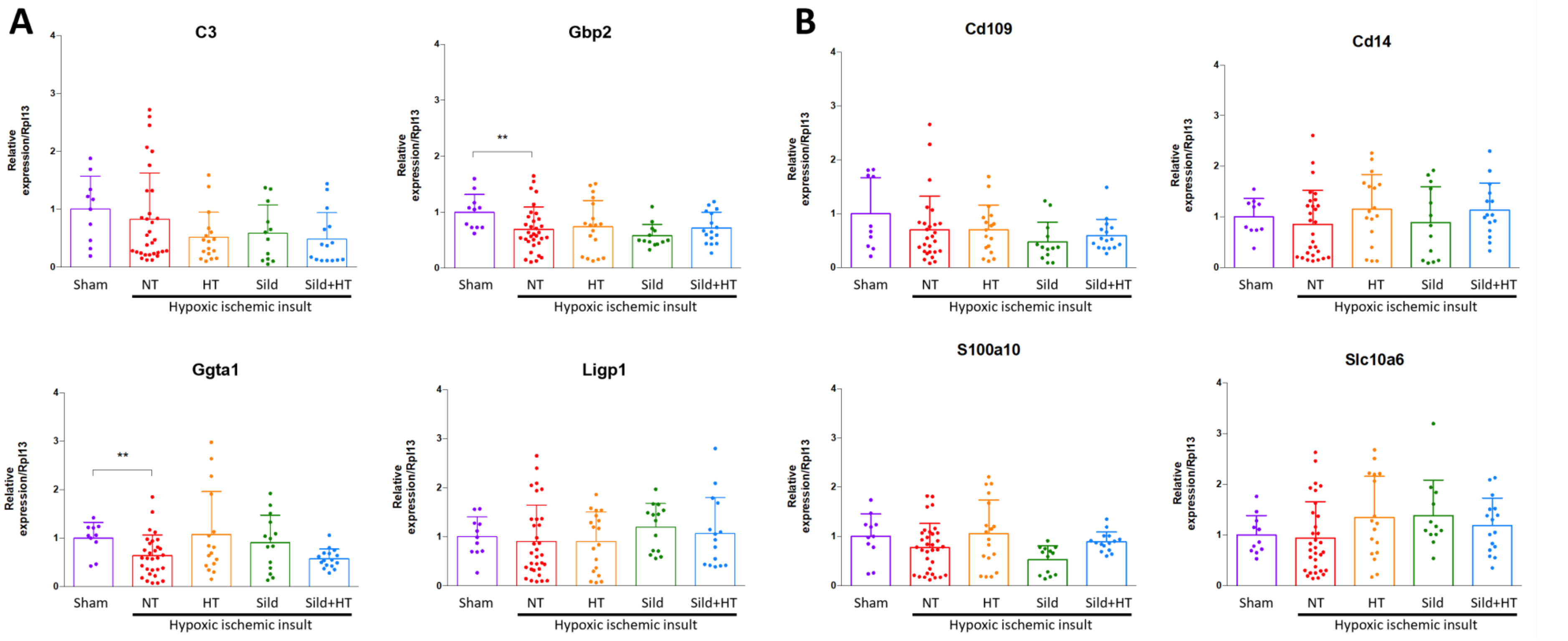

Figure S8: Gene expression polarization in GFAP-positive astrocytes sorted from the contralateral hemisphere of P13 rats subjected to HI at P11 and kept either on normothermia (HI, n=41) compared to sham (n=12), or treated by hypothermia alone (HT, n=19), Sildenafil ip alone (Sild, n=20), and the combined treatment (Sild+HT, n=18). Detailed gene expression of A1 (A) and A2 astrocytic markers (B). Quantified results are mean ± SD. Sham vs untreated (NT) HI animals were first compared using a non-parametric Mann Whitney t-test (**: $p<0.01$). Then, each treatment was compared to untreated animals (HI) using a Kruskal-Wallis test followed by a Dunn's multiple comparison test (all comparisons are not significant).

HI versus Sham

increased genes in HI compared to Sham				decreased genes in HI compared to Sham			
62 prot				82 genes			
Gene Set	Description	P Value	FDR	Gene Set	Description	P Value	FDR
GO:0015850	organic hydroxy compound transport	0.00016977	0.10237	GO:0034612	response to tumor necrosis factor	0.000013007	0.0039818
GO:0006820	anion transport	0.00058586	0.17529	GO:0098754	detoxification	0.000013207	0.0039818
GO:0061024	membrane organization	0.00096955	0.17529	GO:0009617	response to bacterium	0.000036368	0.0073100
GO:0015849	organic acid transport	0.0011628	0.17529	GO:0097237	cellular response to toxic substance	0.00024168	0.036434
				GO:0070555	response to interleukin-1	0.00033296	0.040155
				GO:0050900	leukocyte migration	0.00041856	0.042065
				GO:0001101	response to acid chemical	0.00050503	0.043504
				GO:0042476	odontogenesis	0.0011917	0.081627
				GO:0045087	innate immune response	0.0012183	0.081627
				GO:0046677	response to antibiotic	0.0013845	0.083488
				GO:0052547	regulation of peptidase activity	0.0017749	0.086590
				GO:0098542	defense response to other organism	0.0018514	0.086590
				GO:0006575	cellular modified amino acid metabolic process	0.0018668	0.086590
				GO:0009611	response to wounding	0.0022846	0.091842
				GO:0098609	cell-cell adhesion	0.0022846	0.091842
				GO:0042330	taxis	0.0025567	0.096356
				GO:0007584	response to nutrient	0.0035741	0.12678
				GO:0010038	response to metal ion	0.0051520	0.16493
				GO:0006954	inflammatory response	0.0051967	0.16493
				GO:0071559	response to transforming growth factor beta	0.0072644	0.21902
				GO:0032094	response to food	0.0082701	0.22668
				GO:0071825	protein-lipid complex subunit organization	0.0082701	0.22668

Sild+HT HI versus untreated HI

increased genes in Sild+HT compared to HI				decreased genes in Sild+HT compared to HI			
18 genes				11 genes			
Gene Set	Description	P Value	FDR	Gene Set	Description	P Value	FDR
GO:0009617	response to bacterium	0.0000088987	0.0053659	no pathway			
GO:0009620	response to fungus	0.00037213	0.11220				
GO:0097237	cellular response to toxic substance	0.00091881	0.12213				
GO:0052547	regulation of peptidase activity	0.00098866	0.12213				
GO:0098542	defense response to other organism	0.0010127	0.12213				

Inversely regulated pathways
Aat $logF_C$ >1,3, p-value < 0.05
and FDR<0.25

Figure S9: Over-representation analysis (ORA) using GeneOntology/biological process non redundant of the proteome of MG/M[illegible] cells sorted from the ipsilateral hemisphere of P13 rats subjected to HI at P11 without and with neuroprotective treatment Sild+HT. This ORA analysis was performed to identify the enriched genesets inversely regulated by HI and in HI-injured animals treated by Sild+HT. GO pathways defined by logFC >1,3, p-value < 0.05 and FDR<0.25 were considered significantly up or downregulated. Four genesets were identified as inversely deregulated (highlighted in yellow).

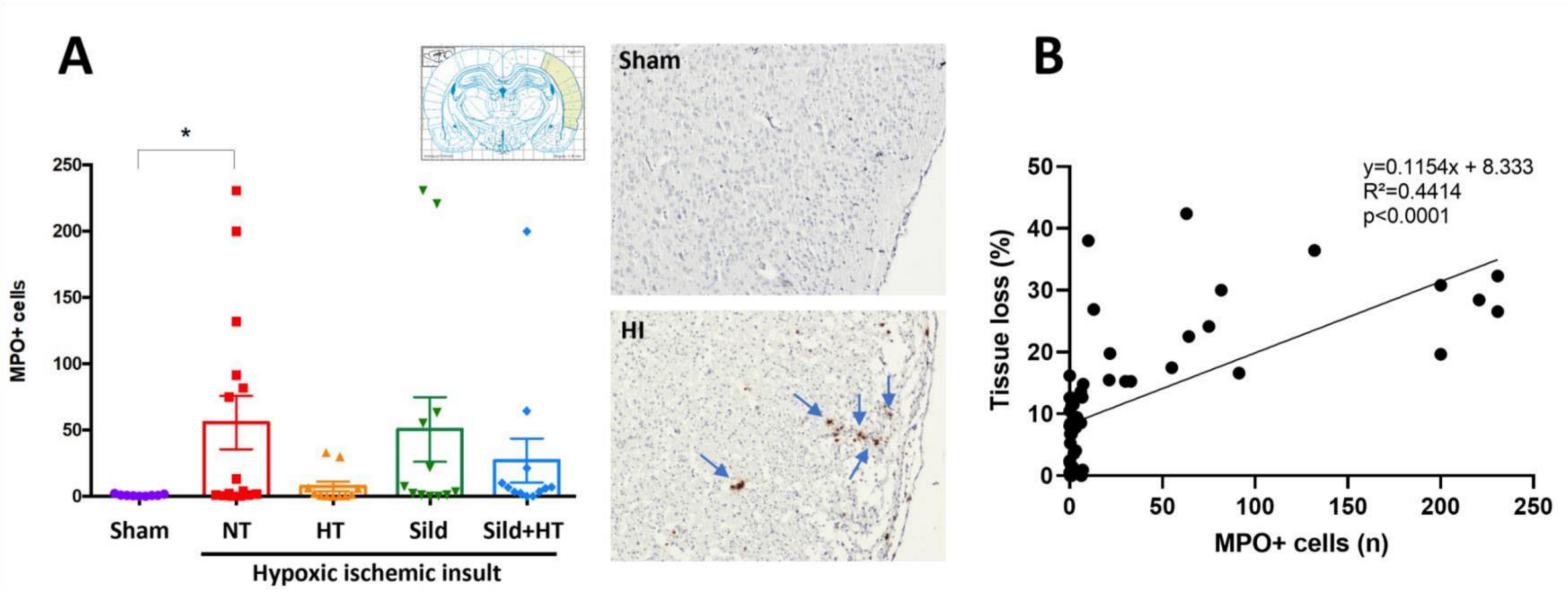

Figure S10: Neutrophilic infiltration in the lesion site.

Quantification of MPO+ cells within S1 cortical brain area, and typical pictures of labelled cells in Sham and HI animals (A). Data are expressed as mean ± SD. Sham vs untreated (NT) HI animals were compared using a non-parametric Mann Whitney t-test (*: $p<0.05$). Linear regression between lesion size and MPO+ cells density in each animals (B).

Table S1 : Primers sequences used for RT-qPCR.

	Microglia		Astrocyte	
	**M1**		**A1**	
pro-inflammatory	Ccl9	GAAGCGTGTAAACTGCCAGC<br>CTGTGACGAGGCTAGGTGTG	C3	GCCTCTACCATGTCGCTACC<br>ATCGAGGATGGTTCAGGGGA
	Cd86	AATCCTTTTCTCGGTGTTGG<br>CTCGGGCTTATGTTTTGAGC	Gbp2	TAAAGGTCCGAGGCCCAAAC<br>AACATATGTGGCTGGGCGAA
	IL1b	CACCTCTCAAGCAGAGCACAG<br>GGGTTCCATGGTGAAGTCAAC	Ggta1	TCTCAGGATCTGGGAGTTGGA<br>GAGTTCTATGGAGCTCCCGC
	IL6	TCCTACCCCAACTTCCAATGCTC<br>TTGGATGGTCTTGGTCCTTAGCC	Ligp1	ATTTGGCTCGAAGCCTTTGC<br>ACGGCATTTGCCAGTCCTTA
	Nos2	AAGAACTCGGGCATACCTTCAG<br>GTCATGAGCAAAGGCACAGAAC		
	Ptgs2	TCC TCC TGT GGC TGA TGA CT<br>CGG GAT GAA CTC TCT CCT CA		
	Tnfa	AAATGGGCTCCCTCTCATCAGTTC<br>TCTGCTTGGTGGTTTGCTACGAC		
	**M2**		**A2**	
anti-inflammatory	Arg1	GGCGTTGACCTTGTCTTGTT<br>GTGATGCCCCAGATGACTTT	Cd14	TCAGAATCTACCGACCATGAAGC<br>GGACACTTTCCTCGTCCTGG
	Mrc1	GACAGACGGACGAGGAGTTC<br>GCCACCAATCACAACAACAC	Cd109	GTCGCTCACAGGTACCTCAA<br>CTGTGAAGTTGAGCGTTGGC
	Il4ra	ACTGGCTGGAACTGTGGTCT<br>CATTGGTGTGGAGTGTGAGG	Slc10a6	TCCATAGAGACCGGAGCACA<br>ATGCCTGATATGCTGCGACA
	Il10	CCTGCTCTTACTGGCTGGAG<br>TTGTCCAGCTGGTCCTTCTT	S100a10	GAAAGGGAGTTCCCTGGGTT<br>CCCACTTTTCCATCTCGGCA
	**reference gene**			
	Rpl13	TCCGAAGAAGGGAGACAGTT<br>CTTCTCCTCTTCCGTGATGG		

Table S2: Analyses of main endpoints according to sex and interaction between sex and each variable analyzed.

Quantified results are mean ± SD or SEM. Sham vs untreated (NT) HI animals were first compared using a non-parametric Mann Whitney t-test (*: p<0.05; **: p<0.01; ***: p<0.001; ****: p<0.0001). Then, each treatment was compared to untreated animals (HI) using a Kruskal-Wallis test followed by a Dunn's multiple comparison test when appropriate (#: p<0.05; ##:p<0.01; ###: p<0.001). Two-way ANOVA was used to assess interaction between sex and treatment.

		**SHAM**		**HI**			**HT**			**SILD**			**SILD + HT**		
		F	M	F	M	Interaction Sham vs HI	F	M	Interaction HT vs HI	F	M	Interaction Sild vs HI	F	M	Interaction SildHT vs HI
**Histology**	**N**	**6**	**3**	**16**	**15**		**8**	**9**		**11**	**12**		**9**	**14**	
Lesion size (cortex)	Mean	0.53	0.62	15.54 ****	18.46 **	P=0.7676	8.47	9.83	P=0.8297	15.01	13.62	P=0.5485	11.79	11.01	P=0.5860
	SD	0.41	1.22	2.53	4.31		2.62	2.75		3.85	3.21		1.76	3.15	
**ICC**	**N**	**6**	**3**	**16**	**15**		**8**	**9**		**11**	**12**		**9**	**14**	
Iba1 (full)	Mean	1.00	1.00	1.81***	2.03**	P=0.4898	1.74	1.42	P=0.1319	1.52	1.80	P=0.6658	1.41	1.38	P=0.2600
	Sem	0.04	0.02	0.14	0.30		0.23	0.12		0.15	0.16		0.10	0.12	
GFAP (full)	Mean	1.26	0.87	1.97	2.38**	P=0.3845	1.45	2.26	P=0.4166	1.67	1.76	P=0.6964	1.49	1.47	P=0.5305
	Sem	0.05	0.04	0.28	0.28		0.21	0.35		0.22	0.27		0.15	0.18	
**qPCR**	**N**	**4**	**6**	**23**	**16**		**8**	**11**		**11**	**10**		**10**	**8**	
Il1b	Mean	0.89	1.08	1.75*	1.51	P=0.4830	1.49	0.83	P=0.5257	1.02	0.98	P=0.5995	0.67$^{\#\#\#}$	0.46$^{\#\#}$	P=0.8324
	Sem	0.20	0.14	0.20	0.27		0.19	0.18		0.13	0.12		0.11	0.05	
Il6	Moy	1.04	0.97	1.15	0.99	P=0.8792	0.85	0.77	P=0.8507	0.77	0.57	P=0.9418	0.27$^{\#\#\#}$	0.37$^{\#\#}$	P=0.5442

	Sem	0.26	0.33	0.19	0.19		0.12	0.17		0.12	0.08		0.03	0.08	
Tnfa	Moy	1.04	0.97	0.95	0.71	P=0.7885	1.10	0.77	P=0.8602	0.35	0.36	P=0.5950	0.60	0.76	P=0.4054
	Sem	0.31	0.12	0.24	0.16		0.17	0.25		0.06	0.08		0.08	0.16	
Cd86	Moy	1.03	0.98	2.04**	2.02**	P=0.9683	2.29	1.82	P=0.3141	1.28$^{\#}$	2.12	**P=0.0418**	1.30$^{\#}$	1.05$^{\#\#}$	P=0.5080
	Sem	0.15	0.20	0.16	0.20		0.24	0.22		0.20	0.23		0.07	0.09	
Nos2	Moy	0.89	1.08	2.93*	2.63	P=0.6691	1.56	1.54	P=0.7900	2.13	1.62	P=0.8524	0.83$^{\#\#}$	0.87$^{\#}$	P=0.7465
	Sem	0.32	0.25	0.46	0.51		0.31	0.29		0.32	0.30		0.14	0.22	
Ptgs2	Moy	1.29	0.81	3.22***	2.70***	P=0.9853	4.08	2.52	P=0.2256	2.33	2.52	P=0.4737	2.01	2.02	P=0.5860
	Sem	0.14	0.07	0.31	0.42		0.49	0.46		0.49	0.47		0.35	0.47	
Arg1	Moy	1.10	0.93	2.28	2.22*	P=0.9143	1.84	1.35	P=0.5770	2.69	1.72	P=0.2823	1.13	1.37	P=0.6882
	Sem	0.21	0.20	0.37	0.33		0.26	0.31		0.65	0.28		0.19	0.27	
Mrc1	Moy	0.93	1.05	0,61*	0.71*	P=0.8963	1.08$^{\#}$	0.80	P=0.1112	0.40	0.62	P=0.4691	0.53	0.57	P=0.7364
	Sem	0.11	0.11	0.06	0.09		0.16	0.18		0.05	0.11		0.06	0.09	
Il10	Moy	0.67	1.22	0.20*	0.34***	**P=0.0570**	0.41	0.47	P=0.6715	0.17	0.20	P=0.3844	0.19	0.30	P=0.8866
	Sem	0.30	0.16	0.04	0.07		0.11	0.15		0.05	0.06		0.06	0.08	
Il4ra	Moy	0.99	1.01	1.51	1.37	P=0.7742	1.72	0.99	P=0.2987	0.74$^{\#\#}$	1.25	P=0.1935	0.90	0.80	P=0.8033
	Sem	0.10	0.14	0.18	0.19		0.11	0.21		0.10	0.19		0.03	0.08	

S3: ORA analyses looking for deregulated biological processes in the HI group compared to the Sham group, depending on various the statistical thresholds.

S/HI	Upregulated genes in HI compared to Sham<br>35 prot				downregulated genes in HI compared to Sham<br>43 genes			
	Gene Set	Description	P Value	FDR	Gene Set	Description	P Value	FDR
P<0,05 et logFc >1,5	GO:0015850	organic hydroxy compound transport	0.00019832	0.11959	GO:0034612	response to tumor necrosis factor	0.0000045705	0.0027560
	GO:0006897	endocytosis	0.00050432	0.15205	GO:0009617	response to bacterium	0.00013414	0.040443
					GO:0098542	defense response to other organism	0.00074791	0.15033
					GO:0045087	innate immune response	0.0010980	0.16552
	62 prot				82 genes			
	Gene Set	Description	P Value	FDR	GO:0034612	response to tumor necrosis factor	0.000013007	0.0039818
	GO:0015850	organic hydroxy compound transport	0.00016977	0.10237	GO:0098754	detoxification	0.000013207	0.0039818
	GO:0006820	anion transport	0.00058586	0.17529	GO:0009617	response to bacterium	0.000036368	0.0073100
	GO:0061024	membrane organization	0.00096955	0.17529	GO:0097237	cellular response to toxic substance	0.00024168	0.036434
	GO:0015849	organic acid transport	0.0011628	0.17529	GO:0070555	response to interleukin-1	0.00033296	0.040155
					GO:0050900	leukocyte migration	0.00041856	0.042065
					GO:0001101	response to acid chemical	0.00050503	0.043504
					GO:0042476	odontogenesis	0.0011917	0.081627
					GO:0045087	innate immune response	0.0012183	0.081627
					GO:0046677	response to antibiotic	0.0013845	0.083488
					GO:0052547	regulation of peptidase activity	0.0017749	0.086590
P<0,05 et logFc >1,3					GO:0098542	defense response to other organism	0.0018514	0.086590
					GO:0006575	cellular modified amino acid metabolic process	0.0018668	0.086590
					GO:0009611	response to wounding	0.0022846	0.091842
					GO:0098609	cell-cell adhesion	0.0022846	0.091842
					GO:0042330	taxis	0.0025567	0.096356
					GO:0007584	response to nutrient	0.0035741	0.12678
					GO:0010038	response to metal ion	0.0051520	0.16493
					GO:0006954	inflammatory response	0.0051967	0.16493
					GO:0071559	response to transforming growth factor beta	0.0072644	0.21902
					GO:0032094	response to food	0.0082701	0.22668
					GO:0071825	protein-lipid complex subunit organization	0.0082701	0.22668

Table

P<0,05 et logFC>0

1229 genes

Gene Set	Description	P Value	FDR
GO:0043043	peptide biosynthetic process	3.0801e-7	0.00018573
GO:0022613	ribonucleoprotein complex biogenesis	0.000076792	0.019676
GO:0006839	mitochondrial transport	0.00011136	0.019676
GO:0006605	protein targeting	0.00013052	0.019676
GO:0051604	protein maturation	0.00022628	0.027289
GO:0009615	response to virus	0.00034018	0.034188
GO:0016197	endosomal transport	0.00042629	0.036722
GO:0055088	lipid homeostasis	0.00050198	0.037837
GO:0090407	organophosphate biosynthetic process	0.00093802	0.059649
GO:0008610	lipid biosynthetic process	0.00098921	0.059649
GO:0140053	mitochondrial gene expression	0.0011186	0.060243
GO:0072348	sulfur compound transport	0.0011989	0.060243
GO:0090662	ATP hydrolysis coupled transmembrane transport	0.0014628	0.067852
GO:0106027	neuron projection organization	0.0020974	0.085506
GO:0072594	establishment of protein localization to organelle	0.0021270	0.085506
GO:0072657	protein localization to membrane	0.0025694	0.092332
GO:0006644	phospholipid metabolic process	0.0027369	0.092332
GO:0019216	regulation of lipid metabolic process	0.0027562	0.092332
GO:0050435	amyloid-beta metabolic process	0.0029388	0.093269
GO:0018209	peptidyl-serine modification	0.0041872	0.12054
GO:0006820	anion transport	0.0041978	0.12054
GO:0071806	protein transmembrane transport	0.0050929	0.13565
GO:0046486	glycerolipid metabolic process	0.0052967	0.13565
GO:0097006	regulation of plasma lipoprotein particle levels	0.0055312	0.13565
GO:0019693	ribose phosphate metabolic process	0.0056406	0.13565
GO:0019882	antigen processing and presentation	0.0058579	0.13565
GO:0007005	mitochondrion organization	0.0062416	0.13565
GO:0015849	organic acid transport	0.0062988	0.13565
GO:0044419	interspecies interaction between organisms	0.0067994	0.14138
GO:0010876	lipid localization	0.0071991	0.14383
GO:0050817	coagulation	0.0073942	0.14383
GO:1901652	response to peptide	0.0077324	0.14571
GO:0001101	response to acid chemical	0.0079826	0.14586
GO:0034660	ncRNA metabolic process	0.0084145	0.14923
GO:0060759	regulation of response to cytokine stimulus	0.0089323	0.15389
GO:0051259	protein complex oligomerization	0.0098561	0.16509
GO:0006753	nucleoside phosphate metabolic process	0.012758	0.20792
GO:0070585	protein localization to mitochondrion	0.013958	0.22149
GO:0042176	regulation of protein catabolic process	0.015621	0.24153
GO:0072511	divalent inorganic cation transport	0.016164	0.24332

838 genes

Gene Set	Description	P Value	FDR
GO:0008380	RNA splicing	1.1956e-12	7.2095e-10
GO:0016071	mRNA metabolic process	1.1298e-11	3.4062e-9
GO:0006403	RNA localization	0.000074397	0.014954
GO:0098754	detoxification	0.00037348	0.056302
GO:0006325	chromatin organization	0.00046827	0.056473
GO:0061458	reproductive system development	0.00078993	0.065797
GO:0042063	gliogenesis	0.00080983	0.065797
GO:0048511	rhythmic process	0.00087293	0.065797
GO:0043631	RNA polyadenylation	0.0018998	0.12728
GO:0015931	nucleobase-containing compound transport	0.0023815	0.14360
GO:0007584	response to nutrient	0.0038390	0.21045
GO:0007568	aging	0.0055323	0.24287
GO:0009617	response to bacterium	0.0055323	0.24287
GO:1901571	fatty acid derivative transport	0.0056388	0.24287

P<0,01 et logFC>0

636 genes

Gene Set	Description	P Value	FDR
GO:0016197	endosomal transport	0.000045398	0.025130
GO:0006605	protein targeting	0.000083350	0.025130
GO:0022613	ribonucleoprotein complex biogenesis	0.00047107	0.079687
GO:0006839	mitochondrial transport	0.00052861	0.079687
GO:0043043	peptide biosynthetic process	0.00075345	0.090866
GO:0051604	protein maturation	0.0010662	0.099945
GO:0019216	regulation of lipid metabolic process	0.0013087	0.099945
GO:0072594	establishment of protein localization to organelle	0.0014625	0.099945
GO:0008610	lipid biosynthetic process	0.0014917	0.099945
GO:0090407	organophosphate biosynthetic process	0.0025451	0.15347
GO:0044419	interspecies interaction between organisms	0.0034567	0.18949
GO:0046486	glycerolipid metabolic process	0.0044899	0.22562

359 genes

Gene Set	Description	P Value	FDR
GO:0008380	RNA splicing	5.1768e-9	0.0000031216
GO:0016071	mRNA metabolic process	0.0000048388	0.0014589
GO:0042063	gliogenesis	0.000074169	0.014908
GO:0009617	response to bacterium	0.00019255	0.029028
GO:0006979	response to oxidative stress	0.00098923	0.11930
GO:0007584	response to nutrient	0.0014919	0.12485
GO:0010038	response to metal ion	0.0016123	0.12485
GO:0042476	odontogenesis	0.0016564	0.12485
GO:0046677	response to antibiotic	0.0021537	0.13338
GO:0010720	positive regulation of cell development	0.0022120	0.13338
GO:0014074	response to purine-containing compound	0.0031400	0.15917
GO:0071407	cellular response to organic cyclic compound	0.0031996	0.15917
GO:0006403	RNA localization	0.0034316	0.15917
GO:0051346	negative regulation of hydrolase activity	0.0039294	0.16214
GO:0061008	hepaticobiliary system development	0.0042852	0.16214
GO:0061458	reproductive system development	0.0043023	0.16214
GO:0032355	response to estradiol	0.0048926	0.16668
GO:0015931	nucleobase-containing compound transport	0.0049754	0.16668
GO:0035690	cellular response to drug	0.0056972	0.18081
GO:0043543	protein acylation	0.0062867	0.18954
GO:0097237	cellular response to toxic substance	0.0070300	0.20186
GO:0048511	rhythmic process	0.0078012	0.20814
GO:0009792	embryo development ending in birth or egg hatching	0.0079392	0.20814

S4: ORA analyses looking for deregulated biological processes in the HI+hypothermia group (HT) compared to the HI untreated group, depending on various the statistical thresholds.

	Upregulated in HT compared to HI				Downregulated in HT compared to HI			
	p<0.05	p<0.05	P<0.05	P<0.01	p<0.05	p<0.05	P<0.05	P<0.01
	logFc>1.5	logFc>1.3	logFc>0	logFc>0	logFc>1.5	logFc>1.3	logFc>0	logFc>0
	6 proteins	13 proteins	165 proteins	28 proteins	2 proteins	3 proteins	402 proteins	70 proteins
**ORA genontology / Biological process no redundant**	NA	ns	ns	ns	NA	NA	ns	ns
**ORA PATHWAY Reactome**	NA	ns	ns	ns	NA	NA	DNA Damage Recognition in GG-NER<br>Regulation of KIT signaling	ns

FDR<0.05
FDR<0.25

Table S5: ORA analyses looking for deregulated biological processes in the HI+Sildenafil group (Sild) compared to the HI untreated group, depending on various the statistical thresholds.

	Upregulated in sild compared to HI				Downregulated in sild compared to HI			
	p<0.05	p<0.05	P<0.05	P<0.01	p<0.05	p<0.05	P<0.05	P<0.01
	logFc>1.5	logFc>1.3	logFc>0	logFc>0	logFc>1.5	logFc>1.3	logFc>0	logFc>0
	4 proteins	7 proteins	268 proteins	58 proteins	8 proteins	11 proteins	467 proteins	127 proteins
**ORA genontology / Biological process no redundant**	NA	NA	organophosphate biosynthetic process	ns	ns	ns	ns	ns
**ORA PATHWAY Reactome**	NA	NA	Purine ribonucleoside monophosphate biosynthesis<br>Nucleobase biosynthesis<br>The phototransduction cascade<br>Visual phototransduction	ns	NA	ns	ns	Interleukin-1 family signaling

FDR<0.05
FDR<0.25

Table

S6: ORA analyses looking for deregulated biological processes in the HI+Hypothermia+Sildenafil group (SildHT) compared to the HI untreated group, depending on various the statistical thresholds.

	Upregulated in sildHT compared to HI				Downregulated in sildHT compared to HI			
	p<0.05	p<0.05	P<0.05	P<0.01	p<0.05	p<0.05	P<0.05	P<0.01
	logFc>1.5	logFc>1.3	logFc>0	logFc>0	logFc>1.5	logFc>1.3	logFc>0	logFc>0
	12 proteins	18 proteins	268 proteins	47 proteins	6 proteins	11 proteins	317 proteins	59 proteins
**ORA genontology / Biological process no redundant**	response to bacterium<br>response to fungus<br>cellular response to toxic substance<br>defense response to other organism<br>detoxification<br>response to antibiotic<br>response to oxidative stress<br>response to interleukin-1	response to bacterium<br>response to fungus<br>cellular response to toxic substance<br>regulation of peptidase activity<br>defense response to other organism	response to oxidative stress<br>small molecule biosynthetic process<br>cellular modified amino acid biosynthetic process<br>cofactor metabolic process<br>detoxification<br>protein complex oligomerization<br>cellular modified amino acid metabolic process<br>adaptive thermogenesis<br>cellular response to toxic substance<br>monocarboxylic acid metabolic process	adaptive thermogenesis	ns	ns	ns	cell junction organization<br>regulation of cell adhesion
**ORA PATHWAY Reactome**	Antimicrobial peptides<br>Neutrophil degranulation<br>Activation of Matrix Metalloproteinases<br>Innate Immune System<br>Degradation of the extracellular matrix	Antimicrobial peptides<br>Neutrophil degranulation<br>Activation of Matrix Metalloproteinases<br>Innate Immune System	Innate Immune System<br>Immune System<br>Neutrophil degranulation	ns	ns	ns	ns	ns

FDR<0.05
FDR<0.25

S7: GSEA analysis against reactome pathways looking for deregulated biological processes in the HI untreated group compared to the Sham group.

Table

	UP
	DOWN

GSEA ANALYSIS AGAINST REACTOME PATHWAY

Gene Set	Description	Size	Leading Edge Number	ES	NES	P Value	FDR
R-RNO-72203	Processing of Capped Intron-Containing Pre-mRNA	176	90	0,70828	2,38	<2.2e-16	<2.2e-16
R-RNO-72163	mRNA Splicing - Major Pathway	136	69	0,73432	2,332	<2.2e-16	<2.2e-16
R-RNO-72172	mRNA Splicing	139	69	0,72493	2,305	<2.2e-16	<2.2e-16
R-RNO-159236	Transport of Mature mRNA derived from an Intron-Containing Transcript	53	29	0,67401	1,922	<2.2e-16	0,0063471
R-RNO-72202	Transport of Mature Transcript to Cytoplasm	53	29	0,67401	1,922	<2.2e-16	0,0063471
R-RNO-72187	mRNA 3'-end processing	45	19	0,7002	1,914	<2.2e-16	0,0052892
R-RNO-1474244	Extracellular matrix organization	63	17	0,6491	1,888	<2.2e-16	0,0090673
R-RNO-73864	RNA Polymerase I Transcription	27	12	0,78201	1,872	<2.2e-16	0,0079339
R-RNO-8875878	MET promotes cell motility	14	6	0,87703	1,861	<2.2e-16	0,011754
R-RNO-109688	Cleavage of Growing Transcript in the Termination Region	50	23	0,66739	1,806	<2.2e-16	0,02308
R-RNO-73856	RNA Polymerase II Transcription Termination	50	23	0,66739	1,806	<2.2e-16	0,02308
R-RNO-73854	RNA Polymerase I Promoter Clearance	26	11	0,75174	1,798	<2.2e-16	0,02292
R-RNO-8943724	Regulation of PTEN gene transcription	29	13	0,75288	1,787	<2.2e-16	0,024412
R-RNO-375165	NCAM signaling for neurite out-growth	15	3	0,81758	1,767	<2.2e-16	0,026446
R-RNO-4551638	SUMOylation of chromatin organization proteins	32	22	0,69012	1,766	<2.2e-16	0,026094
R-RNO-3108214	SUMOylation of DNA damage response and repair proteins	44	27	0,63916	1,755	<2.2e-16	0,026446
R-RNO-73762	RNA Polymerase I Transcription Initiation	24	11	0,75807	1,751	<2.2e-16	0,028002
R-RNO-2990846	SUMOylation	76	27	0,59016	1,734	<2.2e-16	0,031735
R-RNO-1500931	Cell-Cell communication	30	5	0,68627	1,727	<2.2e-16	0,033406
R-RNO-3000171	Non-integrin membrane-ECM interactions	7	3	0,94204	1,724	<2.2e-16	0,032793
R-RNO-6805567	Keratinization	23	7	0,71994	1,699	<2.2e-16	0,043234
R-RNO-74182	Ketone body metabolism	6	3	0,91065	1,691	<2.2e-16	0,045699
R-RNO-3108232	SUMO E3 ligases SUMOylate target proteins	74	26	0,57899	1,678	<2.2e-16	0,052485
R-RNO-5625740	RHO GTPases activate PKNs	18	8	0,74406	1,676	0,025	0,054068
R-RNO-75892	Platelet Adhesion to exposed collagen	5	2	0,9224	1,669	<2.2e-16	0,054351
R-RNO-9018519	Estrogen-dependent gene expression	36	13	0,65068	1,646	<2.2e-16	0,068589
R-RNO-3322077	Glycogen synthesis	5	1	0,92485	1,617	<2.2e-16	0,084922
R-RNO-977606	Regulation of Complement cascade	11	3	0,79669	1,613	0,026316	0,083793
R-RNO-376176	Signaling by ROBO receptors	10	3	0,80472	1,553	<2.2e-16	0,15699
R-RNO-1474228	Degradation of the extracellular matrix	25	5	0,6695	1,552	<2.2e-16	0,15445
R-RNO-75153	Apoptotic execution phase	26	13	0,65895	1,552	<2.2e-16	0,15132
R-RNO-2470946	Cohesin Loading onto Chromatin	8	6	0,81785	1,533	0,021277	0,17595
R-RNO-4085377	SUMOylation of SUMOylation proteins	22	15	0,67033	1,528	0,02381	0,17292
R-RNO-774815	Nucleosome assembly	9	2	0,82107	1,519	<2.2e-16	0,17001
R-RNO-6803529	FGFR2 alternative splicing	15	6	0,78999	1,516	<2.2e-16	0,17
R-RNO-74160	Gene expression (Transcription)	425	95	0,43074	1,514	<2.2e-16	0,16675
R-RNO-9018678	Biosynthesis of specialized proresolving mediators (SPMs)	6	4	0,83142	1,514	0,052632	0,16944
R-RNO-3214858	RMTs methylate histone arginines	21	7	0,69527	1,513	<2.2e-16	0,16591
R-RNO-5685939	HDR through MMEJ (alt-NHEJ)	5	2	0,89126	1,507	0,021277	0,17359
R-RNO-6811555	PI5P Regulates TP53 Acetylation	5	1	0,88191	1,503	0,041667	0,17698
R-RNO-199920	CREB phosphorylation	5	2	0,88164	1,502	0,021739	0,17454
R-RNO-3928663	EPHA-mediated growth cone collapse	5	1	0,86956	1,487	<2.2e-16	0,19185
R-RNO-191273	Cholesterol biosynthesis	12	4	0,69019	1,468	0,027778	0,21348
R-RNO-114508	Effects of PIP2 hydrolysis	12	5	0,71675	1,46	0,054054	0,22501
R-RNO-71064	Lysine catabolism	6	2	0,85237	1,451	0,044444	0,23871
R-RNO-1663150	The activation of arylsulfatases	7	2	0,83612	1,449	0,02381	0,23707
R-RNO-73857	RNA Polymerase II Transcription	368	105	0,42942	1,448	<2.2e-16	0,23621
R-RNO-76042	RNA Polymerase II Transcription Initiation And Promoter Clearance	29	4	0,62904	1,425	0,054054	0,24848

Gene Set	Description	Size	Leading Edge Number	ES	NES	P Value	**FDR**
R-RNO-983712	Ion channel transport	56	26	-0,5956	-1,534	<2.2e-16	0,24026
R-RNO-111931	PKA-mediated phosphorylation of CREB	9	3	-0,8504	-1,539	<2.2e-16	0,23059
R-RNO-8876198	RAB GEFs exchange GTP for GDP on RABs	50	20	-0,6339	-1,567	<2.2e-16	0,15382
R-RNO-2559582	Senescence-Associated Secretory Phenotype (SASP)	26	9	-0,6937	-1,585	0,028986	0,12487
R-RNO-1660661	Sphingolipid de novo biosynthesis	18	8	-0,7454	-1,591	<2.2e-16	0,12586
R-RNO-9007101	Rab regulation of trafficking	66	28	-0,6249	-1,636	<2.2e-16	0,057856
R-RNO-74752	Signaling by Insulin receptor	25	12	-0,7687	-1,697	<2.2e-16	0,026092
R-RNO-163200	iratory electron transport, ATP synthesis by chemiosmotic coupling, and heat production by uncoupling prot	56	17	-0,6567	-1,701	<2.2e-16	0,02712
R-RNO-927802	Nonsense-Mediated Decay (NMD)	41	18	-0,7165	-1,706	<2.2e-16	0,021214
R-RNO-975957	Nonsense Mediated Decay (NMD) enhanced by the Exon Junction Complex (EJC)	41	18	-0,7165	-1,706	<2.2e-16	0,021214
R-RNO-72689	Formation of a pool of free 40S subunits	22	18	-0,8108	-1,711	<2.2e-16	0,022252
R-RNO-156827	L13a-mediated translational silencing of Ceruloplasmin expression	29	20	-0,7563	-1,715	<2.2e-16	0,0225
R-RNO-72649	Translation initiation complex formation	29	20	-0,7563	-1,715	<2.2e-16	0,0225
R-RNO-72662	Activation of the mRNA upon binding of the cap-binding complex and eIFs, and subsequent binding to 43S	29	20	-0,7563	-1,715	<2.2e-16	0,0225
R-RNO-72702	Ribosomal scanning and start codon recognition	29	20	-0,7563	-1,715	<2.2e-16	0,0225
R-RNO-72706	GTP hydrolysis and joining of the 60S ribosomal subunit	29	20	-0,7563	-1,715	<2.2e-16	0,0225
R-RNO-72613	Eukaryotic Translation Initiation	32	23	-0,7643	-1,722	<2.2e-16	0,031683
R-RNO-72737	Cap-dependent Translation Initiation	32	23	-0,7643	-1,722	<2.2e-16	0,031683
R-RNO-72695	Formation of the ternary complex, and subsequently, the 43S complex	25	19	-0,8048	-1,742	<2.2e-16	0,021214
R-RNO-5389840	Mitochondrial translation elongation	68	36	-0,7178	-1,862	<2.2e-16	0,0024107
R-RNO-5368287	Mitochondrial translation	71	36	-0,7134	-1,883	<2.2e-16	<2.2e-16
R-RNO-5419276	Mitochondrial translation termination	70	35	-0,7103	-1,887	<2.2e-16	<2.2e-16
R-RNO-72766	Translation	109	62	-0,7323	-2,034	<2.2e-16	<2.2e-16

S8: GSEA analysis against reactome pathways looking for deregulated biological processes in the HI+Hypothermia group compared to the HI untreated group.

Table

	UP						
	DOWN						
GSEA ANALYSIS AGAINST REACTOME PATHWAY							
No reactome pathway downregulated at FDR<0.25							
Gene Set	Description	Size	Leading Edge Number	ES	NES	**P Value**	FDR
R-RNO-5675482	Regulation of necroptotic cell death	7	2	0,9622	1,76	<2.2e-16	0,1854
R-RNO-5357956	TNFR1-induced NFkappaB signaling pathway	16	2	0,8139	1,759	<2.2e-16	0,1048
R-RNO-75893	TNF signaling	25	6	0,7413	1,739	0,02941	0,0994
R-RNO-5357905	Regulation of TNFR1 signaling	19	3	0,7932	1,68	<2.2e-16	0,2015

Table S9: GSEA analysis against reactome pathways looking for deregulated biological processes in the HI+Sildenafil group compared to the HI untreated group.

No reactome pathway up or downregulated at FDR<0.25

Table S10: GSEA analysis against reactome pathways looking for deregulated biological processes in the HI+Hypothermia+Sildenafil group compared to the HI untreated group.

	UP						
	DOWN						
No reactome pathway downregulated at FDR<0.25							
Gene Set	Description	Size	Leading Edge Number	ES	NES	P Value	**FDR**
R-RNO-389356	CD28 co-stimulation	20	6	0,8661	1,848	<2.2e-16	0,03729
R-RNO-450385	Butyrate Response Factor 1 (BRF1) binds and destabilizes mRNA	12	2	0,8727	1,723	<2.2e-16	0,14917
R-RNO-5218920	VEGFR2 mediated vascular permeability	20	3	0,8095	1,638	<2.2e-16	0,18168
R-RNO-2871796	FCERI mediated MAPK activation	19	3	0,8122	1,632	<2.2e-16	0,18291
R-RNO-5621575	CD209 (DC-SIGN) signaling	15	3	0,8772	1,735	<2.2e-16	0,18646
R-RNO-399954	Sema3A PAK dependent Axon repulsion	10	2	0,9239	1,672	<2.2e-16	0,19268
R-RNO-2682334	EPH-Ephrin signaling	37	9	0,6902	1,638	<2.2e-16	0,19371
R-RNO-450604	KSRP (KHSRP) binds and destabilizes mRNA	9	2	0,892	1,643	<2.2e-16	0,19437
R-RNO-416993	Trafficking of GluR2-containing AMPA receptors	8	4	0,8508	1,618	0,02041	0,19656
R-RNO-199992	trans-Golgi Network Vesicle Budding	46	11	0,6524	1,593	<2.2e-16	0,19837
R-RNO-421837	Clathrin derived vesicle budding	46	11	0,6524	1,593	<2.2e-16	0,19837
R-RNO-6791226	Major pathway of rRNA processing in the nucleolus and cytosol	80	11	0,5759	1,599	<2.2e-16	0,19889
R-RNO-72312	rRNA processing	80	11	0,5759	1,599	<2.2e-16	0,19889
R-RNO-8868773	rRNA processing in the nucleus and cytosol	80	11	0,5759	1,599	<2.2e-16	0,19889

R-RNO-5626467	RHO GTPases activate IQGAPs	19	5	0,7638	1,621	0,03636	0,20221
R-RNO-432722	Golgi Associated Vesicle Biogenesis	35	10	0,6894	1,683	0,02439	0,20386
R-RNO-190840	Microtubule-dependent trafficking of connexons from Golgi to the plasma membrane	10	4	0,8135	1,579	0,01695	0,20599
R-RNO-190861	Gap junction assembly	10	4	0,8135	1,579	0,01695	0,20599
R-RNO-190872	Transport of connexons to the plasma membrane	10	4	0,8135	1,579	0,01695	0,20599
R-RNO-194138	Signaling by VEGF	55	8	0,647	1,654	0,01923	0,20666
R-RNO-5632684	Hedgehog 'on' state	51	12	0,651	1,645	<2.2e-16	0,21008
R-RNO-8856825	Cargo recognition for clathrin-mediated endocytosis	49	17	0,6595	1,658	<2.2e-16	0,2131
R-RNO-4420097	VEGFA-VEGFR2 Pathway	54	8	0,6477	1,689	0,01887	0,21443
R-RNO-5099900	WNT5A-dependent internalization of FZD4	11	5	0,809	1,607	0,03846	0,21616
R-RNO-1433559	Regulation of KIT signaling	7	5	0,8387	1,583	<2.2e-16	0,21828
R-RNO-5696394	DNA Damage Recognition in GG-NER	24	10	0,7592	1,646	0,02128	0,21961
R-RNO-2029480	Fcgamma receptor (FCGR) dependent phagocytosis	48	13	0,6309	1,601	<2.2e-16	0,22375
R-RNO-5358351	Signaling by Hedgehog	71	15	0,607	1,607	<2.2e-16	0,22668
R-RNO-389357	CD28 dependent PI3K/Akt signaling	13	3	0,8291	1,569	<2.2e-16	0,22675
R-RNO-5627123	RHO GTPases activate PAKs	16	3	0,7804	1,559	0,02128	0,24986
